# Simulating Root Growth as a Function of Soil Strength and Yield With a Field-Scale Crop Model Coupled With a 3D Architectural Root Model

**DOI:** 10.3389/fpls.2022.865188

**Published:** 2022-05-20

**Authors:** Sabine Julia Seidel, Thomas Gaiser, Amit Kumar Srivastava, Daniel Leitner, Oliver Schmittmann, Miriam Athmann, Timo Kautz, Julien Guigue, Frank Ewert, Andrea Schnepf

**Affiliations:** ^1^Crop Science, Institute of Crop Science and Resource Conservation, University of Bonn, Bonn, Germany; ^2^Simulationswerkstatt, Linz, Austria; ^3^Institute of Agricultural Engineering, University of Bonn, Bonn, Germany; ^4^Organic Farming and Cropping Systems, University of Kassel, Witzenhausen, Germany; ^5^Albrecht Daniel Thaer-Institut für Agrar- und Gartenbauwissenschaften, Humboldt-Universität zu Berlin, Berlin, Germany; ^6^Chair of Soil Science, TUM School of Life Sciences, Weihenstephan, Germany; ^7^Leibniz Centre for Agricultural Landscape Research (ZALF), Müncheberg, Germany; ^8^Institute for Bio- and Geosciences, IBG-3, Agrosphere, Forschungszentrum Jülich GmbH, Jülich, Germany

**Keywords:** root architecture modeling, subsoil melioration, deep loosening, simulated root length density, root phenotypes, plasticity, *in silico* exploration of GxExM

## Abstract

Accurate prediction of root growth and related resource uptake is crucial to accurately simulate crop growth especially under unfavorable environmental conditions. We coupled a 1D field-scale crop-soil model running in the SIMPLACE modeling framework with the 3D architectural root model CRootbox on a daily time step and implemented a stress function to simulate root elongation as a function of soil bulk density and matric potential. The model was tested with field data collected during two growing seasons of spring barley and winter wheat on Haplic Luvisol. In that experiment, mechanical strip-wise subsoil loosening (30–60 cm) (DL treatment) was tested, and effects on root and shoot growth at the melioration strip as well as in a control treatment were evaluated. At most soil depths, strip-wise deep loosening significantly enhanced observed root length densities (RLDs) of both crops as compared to the control. However, the enhanced root growth had a beneficial effect on crop productivity only in the very dry season in 2018 for spring barley where the observed grain yield at the strip was 18% higher as compared to the control. To understand the underlying processes that led to these yield effects, we simulated spring barley and winter wheat root and shoot growth using the described field data and the model. For comparison, we simulated the scenarios with the simpler 1D conceptual root model. The coupled model showed the ability to simulate the main effects of strip-wise subsoil loosening on root and shoot growth. It was able to simulate the adaptive plasticity of roots to local soil conditions (more and thinner roots in case of dry and loose soil). Additional scenario runs with varying weather conditions were simulated to evaluate the impact of deep loosening on yield under different conditions. The scenarios revealed that higher spring barley yields in DL than in the control occurred in about 50% of the growing seasons. This effect was more pronounced for spring barley than for winter wheat. Different virtual root phenotypes were tested to assess the potential of the coupled model to simulate the effect of varying root traits under different conditions.

## Introduction

Plants require their root systems for multiple reasons, including water and nutrient uptake from soil, and anchoring (Bengough et al., 2006). Specific functions of roots are poorly understood despite their vital roles in crop production and adaptation to stresses (Oyiga et al., 2020). Understanding not only the spatial and temporal distributions of roots but also of the water and nutrients in soil is crucial for root water and nutrient uptake and crop yield. However, root growth in the soil can be affected by the soil's physical, chemical, and biological properties (Bengough et al., 2011). Mechanical impedance due to soil compaction is often considered a major cause of poor growth and development of root systems (Yapa et al., 1988; Bengough et al., 2011; Valentine et al., 2012; Schneider et al., 2017). It is a global problem, a major cause of inadequate root growth and poor crop yields, and a paramount issue for soil management (Batey, 2009; Correa et al., 2019). Compact soils can also be found under natural conditions without human involvement (Batey, 2009). In Germany, compactness of soils is the most common cause for root restriction affecting 51% of crop- and 32% of grasslands (Schneider and Don, 2019). Soil compaction affects not only rooting depth but also infiltration, available water capacity, plant nutrient availability, soil porosity, soil microorganism activity, and thus crop productivity and yield (Busscher, 1990; Batey, 2009; Bengough et al., 2011; Correa et al., 2019). Soil strength, as measured by penetration resistance, varies with a number of soil properties such as soil water content or tension and soil bulk density (Busscher, 1990). Root elongation slows down in drying soil because of a combination of drought stress and mechanical impedance (Batey, 2009; Bengough et al., 2011). Thus, the effect of a given level of compaction is related to weather: large soil moisture deficits can lead to restriction in rooting depth with serious yield effects, while the same degree of compaction can have negligible effects where there are small moisture deficits (Batey, 2009). Correa et al. (2019) reviewed the consequences of soil compaction on root growth. The authors concluded that the role of root system architecture (RSA) plasticity in providing tolerance to soil compaction is complex and poorly understood. They summarized the effects of soil compaction as follows: cases of high resistance to penetration and/or susceptibility of crop genotypes to soil strength would lead to measurable changes in the root system such as (i) reduced root length and number resulting in a smaller root system, (ii) increased root diameter, (iii) less steep root angles, and (iv) deflected root growth (Correa et al., 2019). However, compensatory root growth can be a strategy for adaptive plasticity compensating the limited function of an impaired part of the root system by growing more in looser zones as compared to soil zones with high strength (Goss, 1977; Pfeifer et al., 2014).

The impact of heavy tractors and harvesters on soil compaction can be offset to some extent, e.g., by reducing air pressure in tires; however, soil compaction may still occur in deeper layers (Batey, 2009). Techniques for loosening soil compaction to a depth of 45 cm are well established, but remedying problems at deeper soil depths is still a challenge (Batey, 2009). When roots suddenly encounter a compacted soil layer, e.g., a plow pan, they have three options: (i) they bypass them by distracting themselves sideways, (ii) they penetrate it in order to grow down through the solid soil, or (iii) they stop growing (Clark et al., 2003; Correa et al., 2019). Cultivation of a taproot fodder such as lucerne or chicory enhances the volume of biopores that can be used as “highways” by following crops (Köpke et al., 2015). Either this biological melioration or technical loosening of compacted (sub)soil layers (Jakobs et al., 2017; Sun et al., 2017; Schmittmann et al., 2021) can increase deep rooting and, thus, may enhance yield and/or yield stability especially under dry conditions. Jakobs et al. (2017) and Schmittmann et al. (2021) present a strip-wise loosened subsoil tillage method (30- to 60-cm soil depth) aiming to promote deep rooting by bypassing a clayey soil layer through the loosened strip, and incorporation of different organic materials aimed to stabilize the loosened strip and, thus, avoid soil recompaction. The authors reported that subsoiling combined with compost from biological household wastes increased both root growth and grain yields at the strip.

Process-based dynamic models are still the best quantitative source of our knowledge of plant growth (Stöckle and Kemanian, 2020). They describe the growth of plants in interaction with the environment in a computer language, improve understanding of underlying processes, and allow for scenario analyses. Field-scale crop modeling allows for investigation of soil-root interactions and helps in interpretation of complex data, often non-linear in nature from field trials (Schnepf et al., 2018b; Seidel et al., 2019). Moreover, field trial data can be used to inform and parameterize root architecture models, which can then be used to create realistic scenarios for further investigations (Schnepf et al., 2018a; Morandage et al., 2021a). While soil compaction remains an important factor influencing agriculture, there are only few field-scale simulation studies on how crop yield responds to soil strength and soil loosening. Root simulation models can be distinguished into three-dimensional (3D) functional-structural root architectural models (Dunbabin et al., 2013; Schnepf et al., 2018b) and mostly one dimensional (1D) conceptual simple root models. Accurate modeling of plant reactions to environmental conditions requires information on the spatial geometry of the root system (Mboh et al., 2018). However, many field-scale crop growth models consider simple models to represent the spatial and temporal distributions of roots through 1D vertical density profiles (Williams et al., 1989; Addiscott and Whitmore, 1991; Abrahamsen and Hansen, 2000; Hartmann et al., 2017). Mostly, these models have little feedback from soil properties that may limit root growth (Stöckle and Kemanian, 2020). Only a few studies use field-scale crop models coupled with a root architectural model. Mboh et al. (2018) analyzed the impact of improved RLD simulations due to coupling of a field scale model with a 3D root model on above-ground biomass (AGB) and yield of drought-stressed spring wheat in Germany. Bingham and Wu (2011) tested SPACSYS, a model for carbon and nitrogen cycling in the soil-plant-atmosphere continuum that incorporates a detailed 3D root growth submodel, to predict the growth and RLD distribution of winter wheat. In most root or crop growth models, soil strength dynamics effects on root elongation have not been considered (de Moraes et al., 2018). Kirby and Bengough (2002) combined experiments and simulations of stresses around roots growing in compacted soils to predict the effect of penetration resistance on pea root diameter and elongation rate with a simple model. de Moraes et al. (2018) simulated RLD and root elongation using a stress reduction function. Gaiser et al. (2013) simulated the effect of biopores on root growth and yield of spring crop on a field scale.

Plant (root) phenotypic plasticity in response to climate change and predicted increase of both interseasonal and intraseasonal variability (i.e., frequency and intensity of rainfall, incidences of extreme weather events) may be crucial for maintaining agricultural productivity in the future (Gray and Brady, 2016). The short-term adaptation ability of root systems in response to changing environmental factors could be of great value for site-specific cultivar selection and breeding (O'Toole and Bland, 1987; Kuijken et al., 2015). Fourcaud et al. (2008) concluded that correct simulation of plant growth can only be improved if we correctly understand the relationship among source-sink activity, plasticity, and the environment. Thus, it is important to quantify and assess genotype and environment (G × E) effects when selecting specific site cultivars or developing breeding strategies for improved root traits (Kuijken et al., 2015). Model simulations can support the evaluation of root phene sensitivity to the environment and support novel hypotheses (Postma et al., 2013). Besides, crop models can be used to identify and design future crop ideotypes (Tao et al., 2017).

In our study, we applied a 1D field-scale crop model coupled with a 3D root architecture model to simulate spring barley and winter wheat root and shoot growth based on field data of **two** growth periods per crop cultivated in treatments without (control) and with subsoil (deep) loosening (DL). With the simulation-based approach, we aim to (i) simulate the effect of soil strength on root growth patterns of spring barley and winter wheat, (ii) improve our understanding on how often and under which weather conditions subsoil loosening leads to positive yield effects using weather scenarios, and (iii) elucidate the potential of the coupled model to simulate the effect of different virtual root phenotypes on RLD and yield.

## Materials and Methods

### Experimental Field Data

#### Site Description

The Campus Klein-Altendorf (CKA) Research Facility of the University of Bonn, Germany is located in Rheinbach near Bonn (50°37' 31” N, 6°59' 21” E). The soil on the study site was classified as Haplic Luvisol (Hypereutric, Siltic) derived from loess and is characterized by a silty clay loam texture with clay accumulation in the subsoil between about 45- and 95-cm soil depth. At that site, no pronounced plow pan or subsoil compaction by heavy machinery was observed. The climate at the experimental station can be described as temperate humid with maritime influence. The mean annual air temperature and precipitation (from 2008 to 2020) are 10.5°C and 601 mm (source: homepage CKA), respectively.

#### Experimental Design

Central field experiment 1 (CF1) has a total size of 1.5 ha. The main aim of the experiment was to test the effects of strip-wise subsoil loosening (DL) and, in some treatments (not considered here), the incorporation of an organic material into this strip from 30- to about 60-cm soil depth on crop growth (Jakobs et al., 2019; Schmittmann et al., 2021). The field is subdivided into three trials (CF1-1, CF1-2, and CF1-3) that are all considered in this study. Each plot was replicated three times. Single plot size was 15 m × 3 m (45 m^2^) in CF1-1 and 20 m × 3 m (60 m^2^) in CF1-2. The block-design experiment with the first main crop spring barley (*Hordeum vulgare* L., “Simba”) followed by winter wheat (*Triticumaestivum*, “Desamo”) started in 2017 in CF1-1. In the following year, winter wheat was grown in CF1-1 and spring barley was grown in CF1-2. In 2018/19, winter wheat was grown in CF1-2.

In CF1-3, the intention was to test three different cultivars per crop and treatment. Crop rotation started in 2019 with three spring barley cultivars, Sydney, Eunova, and Salome (sowing date: 29 March 29 2019), which were followed by the three winter wheat cultivars Milaneco, Trebelir, and Capo in 2019/20. The cultivars Sydney, Capo, and Milaneco were selected because of presumed higher root growth in deeper soil layers compared to the other cultivars with more shallow root systems. In this study, we present field data of three growth periods of spring barley (2017, 2018, and 2019) and winter wheat (2017/18, 2018/19, and 2019/20) with focus on the first 2 years (CF1-1 and CF1-2).

#### Soil Preparation and Crop Management

Field site preparation, which was conducted in autumn before sowing of spring barley (2016 in CF1-1, 2017 in CF1-2, and 2018 in CF1-3), included removal of weeds, followed by primary and secondary soil tillage (Supplementary Figure 1). All the plots were tilled using a rotary harrow for seedbed preparation. The deep tilled plots (DL) additionally received subsoil loosening in three steps. In the first step, a strip (or furrow) of 30 cm width and depth was created using a one share plow. A tine worked within the furrow with a target working depth of 60 cm, thus working within a soil depth of 30–60 cm. The strip was placed in the middle of each plot in the direction of tillage and crop management. After this, the soil was reconsolidated using a depth wheel, and the A-horizon was laid back into the furrow using a leveling panel. Regular tillage followed using a rotary harrow for seedbed preparation. After mechanical soil melioration, mustard was sown in autumn and mulched in spring. The field was chisel-plowed (15 cm deep) twice before rotary harrow (10 cm deep) with seedbed preparation took place. The sowing densities were 330 (spring barley) and 300 (winter wheat) seeds per m^2^.

In CF1-1, spring barley was sown on 27 March and harvested on 25 July 2017, and winter wheat was sown on 23 October 2017 and harvested on 17 July 2018. In CF1-2, spring barley was sown on 9 April and harvested on 24 July 2018, and winter wheat was sown on 16 October 2018 and harvested on 8 August 2019. In CF1-3, the three spring barley cultivars were sown 29 March and harvested on 30 July 2020. The three winter wheat cultivars were sown on 18 October 2019 and harvested on 20 July 2020. In all the years, row spacing and within row distances were 12.5 and 3 cm. Spring barley flowering was observed around 12 June 2017, around 18 June 2018, and around 17 June 2019. Winter wheat flowering was observed by the end of May in all the 3 years. A nitrogen (N) fertilizer (calcium ammonium nitrate) was applied based on soil sampling and analysis of topsoil N concentrations. N fertilization on spring barley took place on 17 March 2017 (80 kg ha^−1^), 27 April 2018 (70 kg ha^−1^), and 27 February and 15 April 2019 (50 kg ha^−1^ each). Winter wheat received 50 kg ha^−1^ of N on 27 February and 15 April 2019.According to soil sampling, there was no need for fertilization with phosphorous or potassium. Pests were controlled with pesticides according to standard grower practice.

#### Experimental Data Collection and Statistics

Before the setup of the experiment, soil samples were collected with an auger. Soil texture for different soil depths was determined using these samples. Further soil samples to determine soil bulk density as well as soil organic carbon concentrations were taken in June 2018 down to a depth of 1 m using a sheath probe core sampler (inner diameter 60 mm; Nordmeyer Geotool GmbH) in the control treatments (CF1-1 and CF1-2, 0-1 m) and the DL treatments “at strip” (CF1-1, 0-1m). Total carbon and nitrogen contents were measured by dry combustion (HEKAtechEuroEA 3000). All measurements were performed with at least two analytical replicates. An additional replicate was analyzed when the difference between analytical replicates exceeded 0.5 mg C g^−1^. Inorganic carbon content was quantified using the same technique after mineralization of organic carbon by muffling the samples for 3 h at 550°C. Organic carbon content was determined by subtracting inorganic carbon content to total organic carbon content.

The field data collected during the growth period and presented in this study include mean values of root length density (RLD), shoot biomass, and dry matter grain and straw yield at harvest from the regions of interest “at the strip” of the DL and the control treatment. The RLDs of spring barley were investigated four times in 2017, twice in 2018, and only once for winter wheat around flowering in 2018 and in 2019 applying the profile wall method (Böhm, 1979), a method which is useful to assess relative differences between different treatments with respect to their effect on RLD. However, the method underestimates the absolute RLD as compared to the destructive sampling of monoliths with a defined volume followed by direct measurement of root length. Thus, we used data from monoliths taken in that experiment to convert RLD from the profile wall to absolute RLD.

Around flowering in 2018, the average root diameter of spring barley and winter wheat was observed based on soil samples. For that, the soil monoliths used to convert RLD from profile walls to absolute RLD were soaked in tap water and washed. Subsequently, roots were sorted out by hand, filtering out smallest particles and dead roots, scanned and analyzed with software WinRHIZO version Pro 2017a 64 bit.

During the growth period, biomass samples were cut (0.25 m^−1^ each), dried until constant weight, and weighed. In the melioration treatment, samples were taken both directly above the deep loosening strip and 50 cm next to the melioration strip in an undisturbed area. Dry matter straw yield and grain yield after threshing were determined. Note that part of the observations (RLD from profile wall, yield data) were already published in Jakobs et al. (2019). To test for the significance of differences between the treatments, an analysis of variance (ANOVA, alpha = 0.05) together with Tukey-Kramer *post hoc* test was applied.

## Modeling the Effect of Soil Strength on Root Growth and Yield

### The Modeling Platform SIMPLACE

The 1D field-scale modeling platform Scientific Impact assessment and Modeling PLatform for Advanced Crop and Ecosystem (SIMPLACE) management is written in Java and contains various submodels called SimComponents for simulating the crop growth and development including crop phenology, root growth, soil water dynamics, crop nitrogen, crop water demand, and subsequent abitotic stresses (Enders et al., 2010). A model solution is made by combining different SimComponents depending on research objectives and data availability. The SIMPLACE model solution used in this study contained the following main SimComponents: EvapTranDemand, SlimWater, LintulPhenology, LintulBiomass, LintulWaterStress, and LintulPartitioning. The main SimComponents (www.simplace.net/doc) are described in subsequent sections.

### Evapotranspiration and Soil Water Dynamics

The SimComponent “EvapTranDemand” estimates potential crop transpiration and potential soil evaporation with a modified Penman approach (Penman, 1948, 1956; van Oijen and Leffelaar, 2008). Soil water dynamics are computed by SlimWater (Addiscott and Whitmore, 1991), a routine for transient simulations of the soil water balance of a multiple-layer soil profile. It estimates daily change in soil water content in soil layers based on the volumes of crop water uptake, soil evaporation, surface run-off, and seepage below the root zone. LintulPhenology calculates the development stage of a crop based on the ratio between accumulated growing degree days and the user-defined, crop and cultivar specific temperature sum requirement. LintulBiomass is a generic crop growth model that calculates daily increase in crop total biomass and LAI depending on intercepted radiation and the occurrence of nitrogen or water stress based on the Lintul-2 model (van Oijen and Leffelaar, 2008; Wolf, 2012). Under non-stressed conditions, daily increase in total crop biomass is partitioned into roots, stems, leaves, and storage organs depending on a crop development stage-specific partitioning factor defined in the crop property file.

### Effect of Drought Stress on Partitioning

LintulPartitioning calculates fractions of daily total biomass to be distributed into plant organs, leaves, roots, stems, and storage organs in the SimComponent LintulBiomass. The crop and development stage-specific fractions for the root provided by the user in the partitioning tables in the crop property file are modified daily according to the dominance of drought stress. The daily increase in crop biomass may be reduced by reduction factors for transpiration (TRANRF, Eq. 1). The SimComponent LintulWaterStress (van Oijen and Leffelaar, 2008) calculates transpiration reduction factor (TRANRF) based on the ratio between actual crop transpiration (TRAN in mm) and potential crop transpiration (PTRAN in mm), which are provided by the SimComponent EvapTranDemand:


(1) $$\begin{array}{l} \begin{array}{ll} TRANRF\;=\;MIN(1,\frac{TRAN}{PTRAN}) & \end{array} \end{array}$$

Thus, TRANRF is based on the ratio between actual and potential crop transpirations, which are both calculated by the SimComponent SlimWater. If, at a given day, drought stress is dominant, the fraction of biomass transferred to the roots is increased by multiplying it with the root fraction modification (FRTMOD) factor, which is calculated according to the equation:


(2) $$\begin{array}{l} FRTMOD=MAX\left(1,\frac{1}{TRANRF+0.5}\right), \end{array}$$

where TRANRF is the transpiration reduction factor calculated in the SimComponent LintulWaterStress.

Both factors are dimensionless, TRANRF ranges between 0 and 1 and FRTMOD is equal or >1. The root fraction provided in the partitioning table (FRTTB) is then multiplied with FRTMOD, thereby increasing the amount of assimilates transferred to the roots in the event of moderate to severe drought stress (TRANRF <0.5). Other fractions are then reduced equally to ensure that the sum of all the fractions remains equal to 1. Moreover, the model accounts for the senescence of stems and roots.

The SimComponent SlimRoots, a conceptional root model implemented into SIMPLACE based on Addiscott and Whitmore (1991), estimates the daily increase in seminal and lateral root biomass and converts it into root length per soil layer (in the following: SIMPLACE-SlimRoots). The daily demand of assimilates is limited by crop-specific maximum elongation rate per day (default value: 0.033 m day^−1^). Assimilates provided by the shoot or the seeds are, in the first place, used for the growth of seminal roots, which determine the vertical penetration rate into the soil. In addition to the supply of assimilates by the shoots, the vertical penetration rate of seminal roots depends on soil temperature, soil dryness, and soil strength, which reduce the crop-specific maximum daily elongation rate. If the demand for assimilates by the seminal roots is lower than the supply by the shoots, the remaining assimilates determine the development of lateral roots.

For calculating water uptake in SlimWater, a so-called of root restriction factor (FRR) is calculated considering RLD and root age:


(3) $$\begin{array}{l} FRR\left(i\right)=1-\left(e^{-0.3\;xRDA4AGE\left(i\right)}\right)\text{ for FRR }\leq 1, \end{array}$$

where RDA4AGE depends on the age of the roots and root length density (RLD) in layer i with


(4) $$\begin{array}{l} RDA4AGE\left(i\right)=MAX\left(RLD,RLD\frac{YOUTH}{RLAGE\left(i\right)}\right), \end{array}$$

where RLAGE is the age of the roots in days in layer *i*. YOUTH is the days when roots are considered to be active (for further details, see SlimRoots at https://simplace.net/doc/simplace_modules).

### The 3D Root Architecture Model

CRootbox is a functional-structural root architecture model that simulates root growth according to growth mechanisms including root elongation, branching, orientation, and senescence (Schnepf et al., 2018a,b). Root restriction factor (FRR) is calculated considering RLD but not root age (Eq. 3). Root elongation may be simulated using different growth functions, the two standard ones are linear growth and nonlinear growth in which growth slows down as a root approaches its maximum root length,


(5) $$\begin{array}{l} RL=k(1–\text{exp}(–\frac{re}{k}t)), \end{array}$$

where *RL* is root length (cm), *t* is time (day), *k* is the maximum root length (cm), and *re* is the initial daily root elongation rate (cm day^−1^). This elongation rate may additionally be reduced because of local soil environmental conditions such as high penetration resistance as well as because of carbon availability.

### Root Elongation as a Function of Soil Strength in the Coupled Model

Soil penetration resistance, which varies greatly with soil water status, was modeled as a function of soil water content and bulk density based on a nonlinear soil strength function (Busscher, 1990):


(6) $$\begin{array}{l} Qp=a\gamma^{b}\theta^{c}, \end{array}$$

where *Qp* (MPa) is the soil penetration resistance, γ (Mg m^−3^) is the bulk density, and θ is the volumetric soil water content (cm^3^ cm^−3^). The constants *a, b*, and *c* were set to the default values 0.00587, 8.0772, and −4.65 (Busscher, 1990; de Moraes et al., 2018).

We represent root elongation (RE) as a function of both soil strength (*Q*_*p*_) and matric potential (*h*) for every defined time and soil depth. For simplicity, we assume that these stresses combine linearly (de Moraes et al., 2018), i.e.,


(7) $$\begin{array}{l} RE{\left(Q_{p},\;h\right)}_{t,z}={srf{\left(Q_{p},h\right)}_{t,z}RE}_{\max} \end{array}$$

with


(8) $$\begin{array}{l} srf{\left(Q_{p},h\right)}_{t,z}={\alpha {\left(Q_{p}\right)}_{t,z}\alpha \left(\text{h}\right)}_{t,z}, \end{array}$$

where *srf* (*Q*_*p*_, *h*)_t, z_ is the total stress reduction function for RE due to mechanical (*Q*_*p*_) and hydric (*h*) stresses in each time (*t*) and soil depth (*z*). RE rate can slow down because of soil strength, with an exponential decrease for a soil without continuous macropores. α(*Q*_*p*_) is the stress reduction function by soil strength and is given by Eq. 10 for a soil without continuous macropores (de Moraes et al., 2018):


(9) $$\begin{array}{l} \alpha (Qp)=\exp(xQ_{p}). \end{array}$$

The default value for *x* is −0.4325 (de Moraes et al., 2018).

RE rate, as affected by soil matric potential, *RE*(*h*) in cm day^−1^, is defined as


(10) $$\begin{array}{l} \text{RE}\left(\text{h}\right)=\alpha \left(\text{h}\right)RE_{\max}, \end{array}$$

where α(*h*) is a dimensionless function of soil water pressure head, and *RE*_max_ (cm^−1^) is the maximal possible RE rate without restrictions (de Moraes et al., 2018). Under nonoptimal conditions, i.e., either too dry (water deficit) or too wet (poor aeration), RE is reduced using the stress reduction factor [α(*h*)] from 1 (maximum root elongation) to zero (no growth). The shape of this function for RE follows the concept proposed by Feddes et al. (1978) for root water uptake.

In order to allow for the consideration of treatment-specific soil strength over depth, observed soil bulk densities as well as soil hydraulic parameters estimated on the basis of soil properties including soil bulk density (Table 1) using pedotransfer functions were used in the model.

**Table 1:** Soil properties of the experimental field CF1 (treatments DL and control, mean of CF1-1 to CF1-3) at Campus Klein-Altendorf Research Facility, Rheinbach (University of Bonn, Germany).

**Treatment**	**Depth**	**θFC**	**θred**	**θPWP**	**θS**	**BD**	**Clay**	**Sand**	**Silt**	**SOC**
Control	0–15	0.31	0.22	0.13	0.48	1.3	16.8	8.3	74.9	0.78
Control	15–30	0.3	0.22	0.13	0.42	1.5	16.8	8.3	74.9	0.88
Control	30–45	0.31	0.23	0.16	0.4	1.56	20.4	6.5	73.1	0.59
Control	45–50	0.32	0.25	0.18	0.42	1.5	24.9	7.3	67.8	0.43
Control	50–60	0.32	0.26	0.19	0.41	1.53	26.9	6.7	66.4	0.4
Control	60–70	0.32	0.26	0.2	0.4	1.57	28.1	6.2	65.7	0.37
Control	70–78	0.32	0.26	0.2	0.4	1.59	29.2	6.7	64.1	0.31
Control	78–210	0.32	0.25	0.19	0.39	1.61	27.3	7.9	64.8	0.26
DL	0–15	0.33	0.23	0.13	0.51	1.2	16.8	8.3	74.9	1.06
DL	15–30	0.31	0.22	0.13	0.47	1.32	16.8	8.3	74.9	0.94
DL	30–45	0.32	0.24	0.15	0.47	1.33	20.4	6.5	73.1	0.63
DL	45–50	0.32	0.25	0.17	0.43	1.47	24.9	7.3	67.8	0.41
DL	50–60	0.32	0.26	0.19	0.39	1.59	26.9	6.7	66.4	0.4
DL	60–70	0.32	0.26	0.2	0.4	1.58	28.1	6.2	65.7	0.38
DL	70–78	0.33	0.27	0.2	0.41	1.55	29.2	6.7	64.1	0.41
DL	78–210	0.32	0.26	0.19	0.4	1.58	27.3	7.9	64.8	0.33

*Determined volumetric soil water content (cm^3^ cm^−3^) at field capacity (-33 kPa), θ FC, at which water is available for upward movement (−200 kPa), θred, at permanent wilting point (−1,500 kPa), θPWP, and at saturation (0 kPa), θS. The determined residual water content θres was 0.01%. Observed soil bulk density (BD) (g cm^−3^), clay, sand, silt, and soil organic carbon (SOC) (%) content for the treatments control and deep loosening at the melioration strip (DL at strip). soil depth is given in cm*.

### The Coupling of the SIMPLACE Model Solution and CRootbox

We coupled the SIMPLACE framework and CRootbox with a Python script, running each simulation by daily steps and interchanging the values after each step (in the following: SIMPLACE-cRootbox). Daily root biomass increment from SIMPLACE (LintulBiomass) was converted into maximal daily RE, which is input into CRootbox. The actual RLD derived from CRootbox' simulated root system was input into SIMPLACE. Because of the coupling, the root biomass provided by SIMPLACE determined the maximal root elongation (feedback loop). When potential RE is higher than maximal, CRootbox reduces root growth equally. Decreased root length density may result in decreased soil water and nutrient uptake and increased water/nutrient stress in SIMPLACE, which may reduce total biomass production. Note that potential root growth limitation due to soil physical stresses takes place before potential root growth limitation due to biomass provided by the shoot. In each time step, the amount of carbon potentially available for root growth and the amount of carbon needed for root growth (including limitations due to local soil conditions) are first computed independently from each other. If the available carbon is less than the required carbon, root growth is further restricted by limiting each root equally in its growth until the available and the required carbon demands match. If the required carbon is less than the available carbon, roots can fulfill their growth unlimited from carbon constraints, and excess carbon is absorbed by the shoots. Thus, the simulated actual root length density depends on soil bulk density, soil matrix potential, and C allocated from the shoots to the roots.

### Model Setup, Calibration, and Validation

The model solution of SIMPLACE-CRootbox was used to simulate spring barley (2017 and 2018) and winter wheat (2017/18 and 2018/19) root and shoot growth for two growing periods per crop (CF1-1 and CF1-2). The simulation runs started a few weeks before sowing: on 1 October for winter wheat and on 1 March for spring barley of the respective years. Initial volumetric soil water content was set to 25% (0–30 cm) and 30% (30–210 cm) in spring before sowing of spring barley and to 20% after the very dry summers before sowing of winter wheat. The observed soil texture, soil bulk density, and soil organic carbon content were used to estimate the soil hydraulic parameters using the pedotransfer function HYPRES (Wösten et al., 1999) implemented in SIMPLACE (Table 1). The area of one plant used by CRootbox was estimated based on plant distance and row distance. Growth depression due to nutrient limitation and pests was excluded. The phenology parameters for the SimComponent LintulPhenology (AirTemperatureSumAnthesis, AirTemperatureSumMaturity) and model parameter light use efficiency (LUE) (for parameter descriptions see Supplementary Table 2) were calibrated per crop using the observations: model calibration was based on observed anthesis and harvesting date, grain yield, and AGB from 2017 (spring barley) and 2019 (winter wheat). For model validation, observations of the growing season of 2018 (spring barley and winter wheat) were used. The data set for model calibration, consisting of two seasons per crop, is small, but it is particularly important to correctly simulate the duration from emergence to flowering where root growth occurs. Model calibration was conducted manually and stepwise starting with the phenology parameters AirTemperatureSumAnthesis and then AirTemperatureSumMaturity followed by LUE. The mean absolute error (MAE) and the root mean square error (RMSE) between observed and simulated relevant simulated plant variables were derived with software R version 4.1.2 (R Core Team, 2021).

Since for the cultivar test in CF1-3 (2019/20) field data of only one growth period per cultivar were available, model calibration and validation were not possible. The data of CF1-3 were, thus, used to evaluate the effect of different genotypes on root growth and yield in 2019 (spring barley) and 2019/20 (winter wheat) in both treatments. The model was used to test diverse virtual root phenotypes by changing root parameters systematically (see below) in these two growing seasons. The cultivar trial (CF1-3) was, thus, used to assess the results of the simulation study testing diverse root phenotypes (see below), but the cultivars grown in these years were not simulated explicitly.

The tree parameters r (initial tip elongation rate), ln (length between lateral branches), and lmax (maximal root length) of the default CRootbox root parameters for winter wheat published in Morandage et al. (2019, 2021b) and for spring barley (Eloundou, 2021; Supplementary Table 2) were manually adapted in order to better map RLD and maximum rooting depth. Moreover, we conducted the described simulation runs with the simpler conceptual 1D conceptual root model SlimRoots, which was already implemented into SIMPLACE (SIMPLACE-SlimRoots). All analyses and graphs were made with R.

### Model Runs Under Varying Weather Conditions and for Diverse Root Phenotypes

Model runs under varying weather conditions at that site and, in a second step, for varying cRootbox parameters representing diverse root phenotypes were conducted. The simulation results are not directly comparable to field data but aim to explore the potential of subsoil loosening and the capability of the coupled model to simulate diverse root phenotypes.

To evaluate the effect of subsoil loosening under various weather conditions, simulation runs for both treatments using the SIMPLACE-CRootbox model were conducted. For that, the 12 years where daily weather data were available from the CKA Research Facility (2008–2019) were applied.

Oyiga et al. (2020) evaluated the root responses of 192 spring barley genotypes to water shortage at the same research facility (CKA) and reported a wide range of root architectural traits and root phenotypic diversity. To explore the capability of the coupled model to simulate diverse root phenotypes, we varied the CRootbox parameters ln (length between lateral branches), maxB (maximal number of basal roots), and r (initial tip elongation rate) for the weather conditions in 2019 (spring barley) and 2019/20 (winter wheat), and evaluated the simulated RLD and yield.

All scenario simulations started on 1 March (spring barley) and 1 October (winter wheat) each year with the same initial conditions as described above and in Table 1. The sowing dates were set to 27 March (spring barley) and 27 October (winter wheat) each year.

## Results

The strip-wise subsoil loosening in treatment DL reduced soil bulk density by about 16% (in 30- to 45-cm soil depth) compared to the control treatment (Table 1). Note that part of the observed RLD and yield data was published and discussed in detail in Jakobs et al. (2019) and Schmittmann et al. (2021).

### Field Observations

#### Growing Conditions and Crop Development

At the Campus Klein-Altendorf Research Facility (University of Bonn, Germany), the season in 2017 can be characterized as a normal growth period with abundant precipitation, whereas the main spring and summer growth period in 2018 was very dry, and in 2019 it was dry and very hot (Supplementary Figure 2). The cumulative rainfall during the spring barley growth periods was 249 mm in 2017 but only 163 mm in 2018 and 154 mm in 2019. The soil was extremely dry and hard weeks before harvest in 2018, and plants suffered from heat and drought stress. The precipitation in the spring and summer seasons in 2019 and 2020 was also below average (about 200 and 146 mm from March to the end of June). In 2019, a very hot period occurred from mid June to mid July with daily mean air temperatures of up to 35°C (25 June).

#### Observed Root Growth

**Figure 1:**
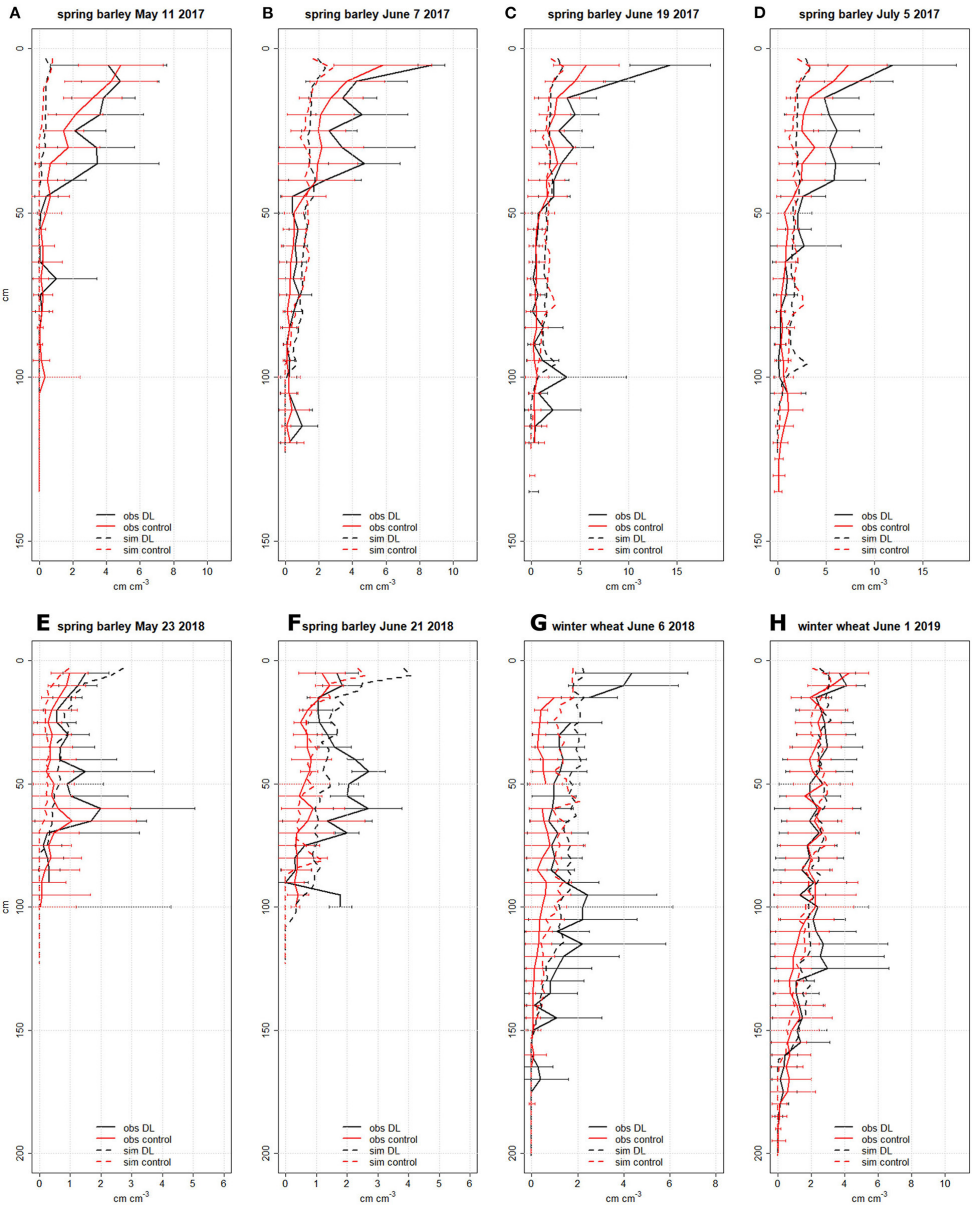
Observed (mean and standard deviation) and simulated (SIMPLACE-CRootbox) absolute root length densities (RLDs) over soil depth in cm cm^−3^ of spring barley in 2017 (top panel **[A–D]**) and 2018 (bottom panel **[E,F]**) and winter wheat (2018 and 2019, bottom panel **[G,H]**) for the control and deep loosening (DL) (at strip) treatments. The RLD data were observed at profile walls and converted to absolute values based on profile wall and monolith data. The RLD data observed with the profile wall method were partly published in Jakobs et al. (2019).

In general, the strip-wise deep loosening fostered higher RLD of spring barley and winter wheat in most soil depths at the strip (DL treatment) as compared to the control treatment (Figure 1). The soil depth where root growth was mainly enhanced in DL compared to control varied over the years. In the very dry growing season in 2018, the deep loosening especially enhanced RLD in the topsoil (0–30 cm) and the loosened soil layer (30–60 cm) (Figures 1E–G). In spring barley in 2017, RLD was mainly enhanced in the topsoil whereas in 2018, RLD was mainly enhanced in about 30–80 cm soil depth. Similar to shoot biomass (see below), absolute RLD was higher in the growth periods with normal rainfall pattern (2017 and 2019) than in the very dry year 2018. In 2017, the observed topsoil RLD of spring barley was higher in treatment DL compared to the control, which was especially pronounced in June. In the deep soil below 90 cm soil depth, more roots were found at DL as compared to the control on June 19. The maximal rooting depth of spring barley observed on 5 June 2017 was 105 cm (DL) and 135 cm (control). In 2018, the RLD of spring barley observed at the deepest soil depth was higher in treatment DL than in the control, but at the sampling date on 21 June, the maximal sampling depth was 100 cm. In 2018, spring barley RLD was much higher in treatment DL than in the control in most soil depths and on both dates. In 2018, winter wheat RLD in treatment DL was increased in all soil depths compared to the control treatment. In 2019, winter wheat RLD was similar in treatments DL and control until about 100-cm soil depth, with higher RLD from about 100–150 cm in DL as compared to the control treatment. The maximal observed rooting depth of winter wheat was lower in the DL than in the control treatment (170 vs. 180 cm in 2018 and 185 vs. 195 cm in 2019).

In CF1-3, the observed RLD from 0 to 30 cm was highest for the shallow rooting spring barley cultivar Salome, whereas from 30- to 100-cm soil depth, it was highest for the deep rooting cultivar Eunova (control and DL treatment at flowering, not shown). Furthermore, the RLD observed at flowering was higher in DL than in the control, especially from about 20 to 70 cm soil depth. In 2020, about 30% lower RLD below 30 cm and, in particular, from 60 to 180 cm soil depth of the shallow rooting winter wheat cultivar Trebelir compared to the deeper rooting cultivars Milaneco and Capo was observed (control treatment at flowering, not shown).

#### Observed Above-Ground Biomass and Grain Yield

**Figure 2:**
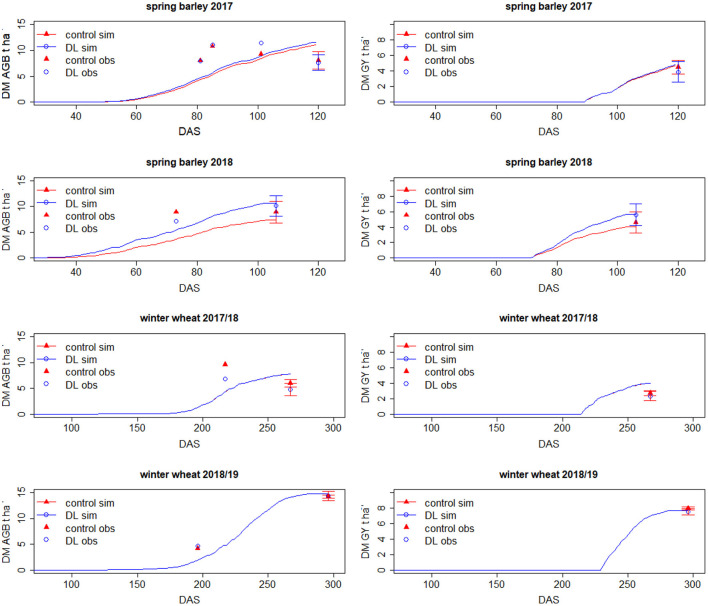
Observed (dots and triangles) and simulated (lines, applying SIMPLACE-CRootbox model) dry matter above-ground biomass during the growth season and at harvest (DM AGB) and grain yield (DM GY) in t ha^−1^ of spring barley (2017 and 2018) and winter wheat (2017/18 and 2018/19) for the control and DL (at strip) treatments. The observed yield data at harvest are given as mean values with standard deviation.

In 2018, winter wheat yield level was very low mainly because of the drought, which caused emergency ripening (Table 2). In all the growth seasons, differences in yield between the control and DL treatments were not significant, with a tendency for higher grain yield and AGB in the control than in the DL (at strip) treatment (Figure 2). Only in the very dry season of 2018 spring barley yielded 18% (but non-significantly) higher in treatment DL, with 5.6 compared to 4.6 t ha^−1^ in the control treatment. Thus, for the spring cereal cultivated in 2018, the enhanced RLD due to strip-wise deep loosening paid off.

**Table 2:** Observed (obs, mean ± standard deviation) and simulated spring barley (cultivar “Simba”) and winter wheat (cultivar “Desamo”) dry matter grain yield and above-ground biomass, both in t ha^−1^, in 2017, 2018, and 2019 for the treatment with strip-wise subsoil loosening (DL) at the melioration strip and the control treatment (CF1-1, CF1-2).

**Year**	**Treatment**	**Crop**	**Grain yield**			**Above-ground biomass**		
			**obs**	**CRootbox**	**SlimRoots**	**obs**	**CRootbox**	**SlimRoots**
2017	DL	Spring barley	3.9 ± 1.3	4.8	4.8	7.6 ± 1.5	11.6	11.8
2017	control	Spring barley	4.5 ± 0.9	4.6	4.8	8.1 ± 1.7	11.0	11.8
2018	DL	Spring barley	5.6 ± 1.0	5.5	6.2	10.1 ± 2.0	10.7	12.2
2018	control	Spring barley	4.6 ± 1.4	4.0	6.1	8.9 ± 2.1	7.3	12.1
2017/18	DL	Winterwheat	2.3 ± 0.6	4.0	4.1	4.8 ± 1.2	7.7	7.9
2017/18	control	Winterwheat	2.7 ± 0.3	4.0	4.1	6.0 ± 0.7	7.7	7.9
2018/19	DL	Winterwheat	7.5 ± 0.4	7.7	7.7	14.4 ± 0.9	14.8	14.9
2018/19	control	Winterwheat	8.0 ± 0.2	7.7	7.7	14.3 ± 0.3	14.8	14.9

*Simulations were conducted with a SIMPLACE solution coupled with a 3D architectural root model CRootbox (SIMPLACE-CRootbox) and with a SIMPLACE solution with a conceptional 1D root model SlimRoots (SIMPLACE-SlimRoots), details see manuscript. No significant differences (ANOVA, alpha = 0.05) of yield and AGB between the treatments per year and crop were observed*.

In CF1-3 where three cultivars with presumed differences in deep rooting characteristics were tested, grain yield did not differ significantly: mean spring barley grain yield was 4.8 (DL) and 4.9 t ha^−1^ (control), and mean winter wheat grain yield was 6.4 (DL) and 6.2 t ha^−1^ (control) (Table 3). The deep rooting spring barley cultivar Eunova reached lower yields in the DL treatment compared to the control (4.5 vs. 4.9 t ha^−1^) in 2019, but the difference was not statistically significant. Similarly, the deep rooting winter wheat cultivar Milaneco had lower yield in the DL treatment compared to the control (6.8 vs. 7 t ha^−1^) in 2020, but the difference was, again, not significant. The Grain yield of the deep rooting winter wheat cultivar Capo was 14% higher (nonsignificant) in the DL treatment as compared to the control (6.9 vs. 5.9 t ha^−1^). Thus, there was no clear trend for an impact of deep loosening on yield and AGB of deep or shallow rooting cereal cultivars in 2019/20.

**Table 3:** Observed (obs, mean ± standard deviation) spring barley and winter wheat dry matter grain yield and AGB, both in t ha^−1^, in 2019 for three cultivars of the treatment with strip-wise subsoil loosening (DL) at the melioration strip and the control treatment (CF1-3).

**Year (field)**	**Crop (cultivar)**	**Grain yield**	**above-ground biomass**
**treatment**			
**2019 (CF1-3)**
DL	barley (Sydney)	5.1 ± 0.4	9.6 ± 0.4
DL	barley (Eunova)	4.5 ± 0.4	8.4 ± 0.6
DL	barley (Salome)	4.8 ± 0.9	9.0 ± 0.7
control	barley (Sydney)	5.1 ± 1.1	9.6 ± 1.1
control	barley (Eunova)	4.9 ± 0.7	9.7 ± 0.7
control	barley (Salome)	4.7 ± 0.9	8.8 ± 0.9
**2019/20 (CF1-3)**
DL	wheat (Milaneco)	6.8 ± 0.9	16.0 ± 0.7a
DL	wheat (Trebelir)	5.7 ± 0.9	14.6 ± 1.3ab
DL	wheat (Capo	6.9 ± 0.5	15.3 ± 1.0ab
control	wheat (Milaneco)	7.0 ± 0.5	15.1 ± 1.0ab
control	wheat (Trebelir)	5.9 ± 0.4	14.6 ± 2.4ab
control	wheat (Capo)	5.9 ± 0.4	12.0 ± 1.1c

*Different letters indicate significant differences between the groups (observations) per year and crop (ANOVA, alpha = 0.05)*.

### Modeling Results

#### Model Calibration and Validation

Two phenology model parameters were fitted to the cultivar anthesis and harvest dates of spring barley 2017 and winter wheat 2017/18 (Supplementary Table 2). The parameter x in the soil strength factor equation (Eq. 8) was fitted according to the observed differences in RLD between DL at strip and the control and set to −0.0025 (spring barley) and −0.005 (winter wheat). Mean specific root weight was set to 0.00002 g cm^−1^. Three CRootbox parameters (r, ln, and lmax) were fitted to better represent the observed root growth dynamics over depth and time (see Supplementary Table 2).

#### Simulated Root and Shoot Growth Using the SIMPLACE-SlimRoots Model

Using the simpler conceptual root model (SIMPLACE-SlimRoots), the enhanced absolute RLD in the DL treatment compared to the control was hardly reflected (Supplementary Figure 3). In 2017, slightly higher spring barley RLD at 50 cm soil depth was simulated. In 2018, spring barley RLD at the top layer was slightly higher in the DL than in the control treatment. Note that the RLD from 45 to 168 cm in 2018 is 0.1 cm cm^−3^, which is barely visible in Supplementary Figure 3. This enhanced deep rooting led to a simulated spring barley grain yield increase of 0.1 t ha^−1^ for DL compared to the control (Table 2). The simulated grain yields did not differ between the treatments except for spring barley in 2018. The simulated maximal rooting depth of spring barley was 165 cm (DL) and 162 cm (control) in 2017 and 168 cm in 2018. In winter wheat, the simulated maximal rooting depth was 210 cm in 2018 and 2019 (both treatments).

#### Simulated Root and Shoot Growth Using the SIMPLACE-CRootbox Model

##### Root Length Density and Maximal Rooting Depth

In general, the observed and simulated absolute RLD values point in a similar direction (Figure 1). Exceptions are spring barley in 2017 where the observed values in the upper layers were higher than in the simulated ones and for spring barley in 2018 (DL) where the observed subsoil RLDs were higher than the simulated ones. The enhanced absolute RLD in the DL treatment as compared to the control was predicted with the SIMPLACE-CRootbox model in most cases, however, to a lesser extent than observed (Figure 1). For example, on 5 July 5 2017 (Figure 1D), the observed spring barley RLD from 0 to 60 cm soil depth differed by about 50%, whereas the model predicted only 10% higher RLD for DL as compared to the control. The mean winter wheat RLD difference between both treatments from 0 to 150 cm soil depth in June was 60% (observed) vs. 30% (simulated) in 2018 and 10% (observed) vs. 15% (simulated) in 2019. The enhanced observed and simulated RLDs in the DL treatment compared to the control is especially prominent in the dry growth period of spring barley in 2018 (Figures 1E,F). Similar to the observations, the simulated treatment effect was minor for winter wheat in 2018/19 (Figure 1H). The simulated cumulated RLD over depth (in 3-cm layer discretization) at flowering differed in particular in 2018 between both treatments with 47 (DL) vs. 25 cm cm^−3^ (control) for spring barley (Figure 1F) and with 69 (DL) vs. 49 cm cm^−3^ (control) for winter wheat (Figure 1G). In 2017, the simulated spring barley RLD over soil depth was similar for both treatments until about day after sowing (DAS) 50, but it differed from the beginning in 2018, which explains the treatment differences in 2018 (Supplementary Figure 5). In 2018, the simulated cumulative spring barley water uptake was 124 mm (DL) and 81 mm (control) (Supplementary Figure 6). In that season, the enhanced root growth in the early season and the deeper rooting fostered, in particular, spring wheat water uptake in upper soil layers in the early season as well as water uptake in the lowest soil layers in treatment DL as compared to the control (Supplementary Figures 6E,F). The maximal simulated rooting depth of spring barley was 111 cm (DL) and 127 cm (control) in 2017 as well as 108 cm (DL) and 84 cm (control) in 2018. In 2017/18, the maximal simulated rooting depth of winter wheat was 153 cm (DL) and 156 cm (control), and in 2018/19 it was 162 cm (DL) and 168 cm (control). The observed higher RLDs in the very deep soil layers around the 170 cm soil depth of winter wheat in 2018/19 in the control treatment compared to the DL treatment were simulated. In the dry growing season of 2018, the observed and simulated higher RLDs in the deep soil layers of spring barley (observed until 1 m) were higher in the DL treatment than in the control.

##### Above-Ground Biomass and Grain Yield

In general, the prediction of absolute yield was accurate, but the sharp winter wheat yield decline in 2018 due to the dry spell was underestimated by the model (Table 2). For the growing periods of 2017 and 2019 (calibration), the MAE and RMSE for grain yield were 0.4 and 0.3 t ha^−1^ and for AGB 2 and 2.5 t ha^−1^. For the growing period of 2018 (data used for validation, see Table 2), the MAE and RMSE for grain yield were 1.2 and 1 t ha^−1^ and for AGB 1.9 and 1.7 t ha^−1^. A difference in grain yield and AGB between both treatments was simulated only for spring barley (Table 2). Especially in 2018, the simulated grain yield was higher in the treatment with deep loosening compared to the control (5.5 vs. 4 t ha^−1^). Besides, simulated spring barley AGB and yield were slightly higher in 2017 in the DL treatment than in the control. For winter wheat, no differences in grain yield and AGB between the treatments were simulated. In 2017, higher spring barley RLD in the DL treatment than in the control between the 50th day after sowing and flowering was simulated (Supplementary Figure 6). This difference almost disappeared at flowering, leading to a slightly lower grain yield in the control than in the DL treatment. In contrast, simulated spring barley shoot and root biomass and RLD were enhanced in DL in 2018 from early crop development stages onward (Supplementary Figures 5, 6). A yield decline due to deep loosing was not simulated.

#### Prediction Capability of the Effect of Subsoil Loosening on Cereal Growth Pattern

In general, the main growth pattern of the cereals in the 3 years was simulated by the coupled model (Table 4). Due to the research question focusing on the effect of soil strength on root growth, the focus was on the prediction of the root growth pattern.

**Table 4:** Observed growth pattern for 2017, 2019 (growth periods with low to normal rainfall), and 2018 (extremely dry growing period), and prediction of the respective pattern for the treatment with strip-wise subsoil loosening (DL) at the melioration strip and the control treatment (✓: pattern was predicted, (✓): pattern was partly predicted, and X: pattern was not predicted).

**Observed pattern**	**Predicted**
**Extremely dry growing season (2018)**	
Enhanced spring barley RLD in DL	✓
Enhanced topsoil winter wheat RLD in DL	✓
Enhanced subsoil winter wheat RLD in DL	✓
Enhanced deep subsoil winter wheat RLD and maximal rooting depth in DL	✓
Tendency for higher spring barley yields in DL (18%)^T^	✓
Tendency for lower winter wheat yields in DL (14%)^T^	X
**normal to low rainfall (2017, 2019)**	
Enhanced topsoil spring barley RLD in DL	(✓)
Enhanced deep subsoil spring barley RLD and maximal rooting depth in control	(✓)
Enhanced topsoil winter wheat RLD in DL	✓
Enhanced deep subsoil winter wheat RLD and maximal rooting depth in control	✓
Tendency for lower spring barleyyields in DL (13%)^T^	X
Similar winter wheat yield and AGB in both treatments	✓

*Simulations were conducted with a SIMPLACE solution coupled with a 3D architectural root model CRootbox (SIMPLACE-CRootbox), for details see the main manuscript. ^T^No significant differences between the groups (observations) per year and crop were observed (ANOVA, alpha = 0.05)*.

#### Model Runs Under Varying Weather Conditions

**Figure 3:**
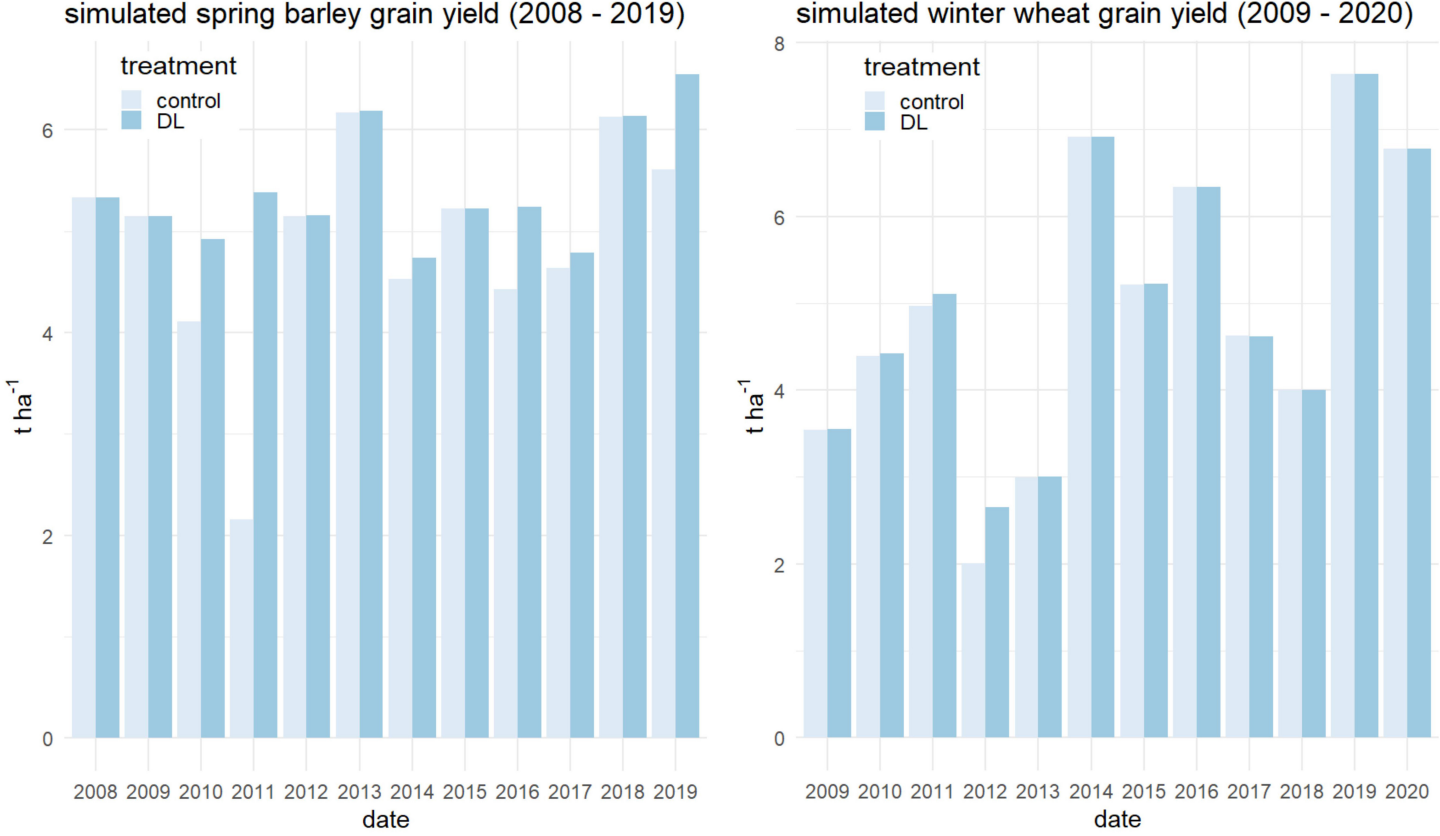
Simulated spring barley (left) and winter wheat (right) grain yield in t ha^−1^ for the harvesting years 2008 to 2020 at the CKA Research Station, Germany using the coupled SIMPLACE-cRootbox model.

In six out of twelve growing seasons, a yield increment due to deep loosening was simulated (Figure 3). The mean spring barley grain yield of treatment DL was 10% higher than that of the control. In 2010, 2016, and 2019, the grain yield increase due to deep loosening was about 15%. In these growing seasons, spring barley root and shoot growth was slower in the early developmental stages in the control than in the DL treatment because of dry topsoil conditions. In 2010, 2011, and 2019, precipitation was very low in April (8 mm in 2010, 22 mm in 2011, and 24 mm in 2019 vs. a mean precipitation from 2008 to 2019 of 34 mm). In 2016, with above-average rainfall in April (31 mm), crop emergence and rooting into the topsoil took place about two weeks later in the case of the control compared to the DL treatment (Supplementary Figure 8). In 2011, the yield increase due to deep loosening was even 60% and caused by very dry topsoil in early spring, which hampered root elongation especially in the control treatment (Supplementary Figure 8). In the other years, rooting into the topsoil at the beginning of the growing season was also a few days faster in DL than in the control treatment, but the reduced RE at the beginning was compensated by plant water uptake during the growth period and did not lead to yield decline.

In the case of winter wheat, the low initial soil moisture at sowing imitating a very dry summer resulted in rather low winter wheat yield level in several years (mean grain yield: 5 t ha^−1^) for that site. The mean yield gain due to deep loosening was only 2% with an impact on grain yield in four out of the 12 years. Only in one out of the 12 years, namely in the growth period of 2011/12, a yield gain due to deep loosening of 24% was achieved. In this season, maximal rooting depth in the end of March was higher for DL than for the control (87 vs. 72 cm). In that season, only about 22% of the mean precipitation in March was observed, which retarded rooting in spring in the control treatment. Thus, in years with very dry spring periods, deep loosening has paid off.

The weather scenarios were also tested with the simpler root model (SIMPLACE-SlimRoots). Here, the mean simulated grain yield of winter wheat and spring barley was 5.1 and 5.4 t ha^−1^. The simulated mean grain yield difference for both treatments was about 1% for both crops and, thus, lower than for the coupled model.

#### Simulation of Different Virtual Root Phenotypes

Oyiga et al. (2020) reported substantial genotypic variability for root architectural traits of spring barley, including the number of nodal roots that emerged at the main shoot axis and tillers.

**Figure 4:**
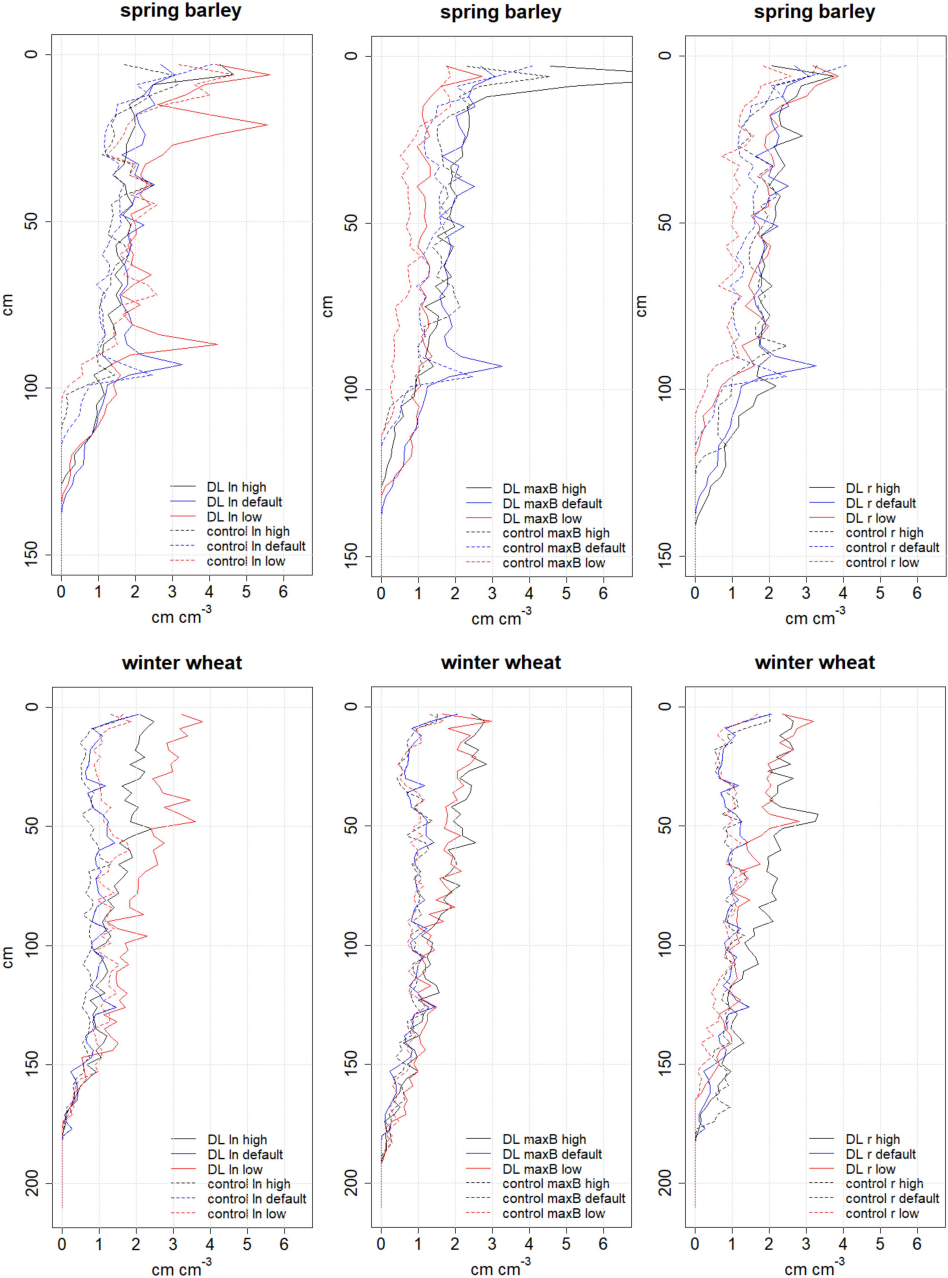
Simulated RLD in cm cm^−3^ at flowering of spring barley (top panel) in 2019 and winter wheat (bottom panel) in 2019/20 for phenotypic diversity of root traits using the coupled SIMPLACE-CRootbox model. Applied parameters: ln: length between lateral branches in cm (wheat: low: 1.5, default: 2, and high: 2.5; barley: low: 0.65, default: 0.85, and high: 1); maxB: maximal number of basal roots (wheat: low: 10, default: 20, and high: 30; barley: low: 2, default: 5, and high: 7), and r: initial tip elongation rate (wheat and barley: low: 5, default: 7, and high: 9). For root nomenclature, see Zobel and Waisel (2010).

We varied three CRootbox parameters for testing the effect of different virtual but plausible phenotypes. Hereby we first increased and then decreased the parameter value of each parameter in sequence while keeping the other parameters constant and evaluated the simulated RLD and yield. Figure 4 shows the effects of phenotypic diversity on simulated RLD distribution over soil depth for 2019 and 2019/20. The coupled model was, in principle, able to represent the behavior of different genotypes including RLD and the feedback on biomass and yield.

**Figure 5:**
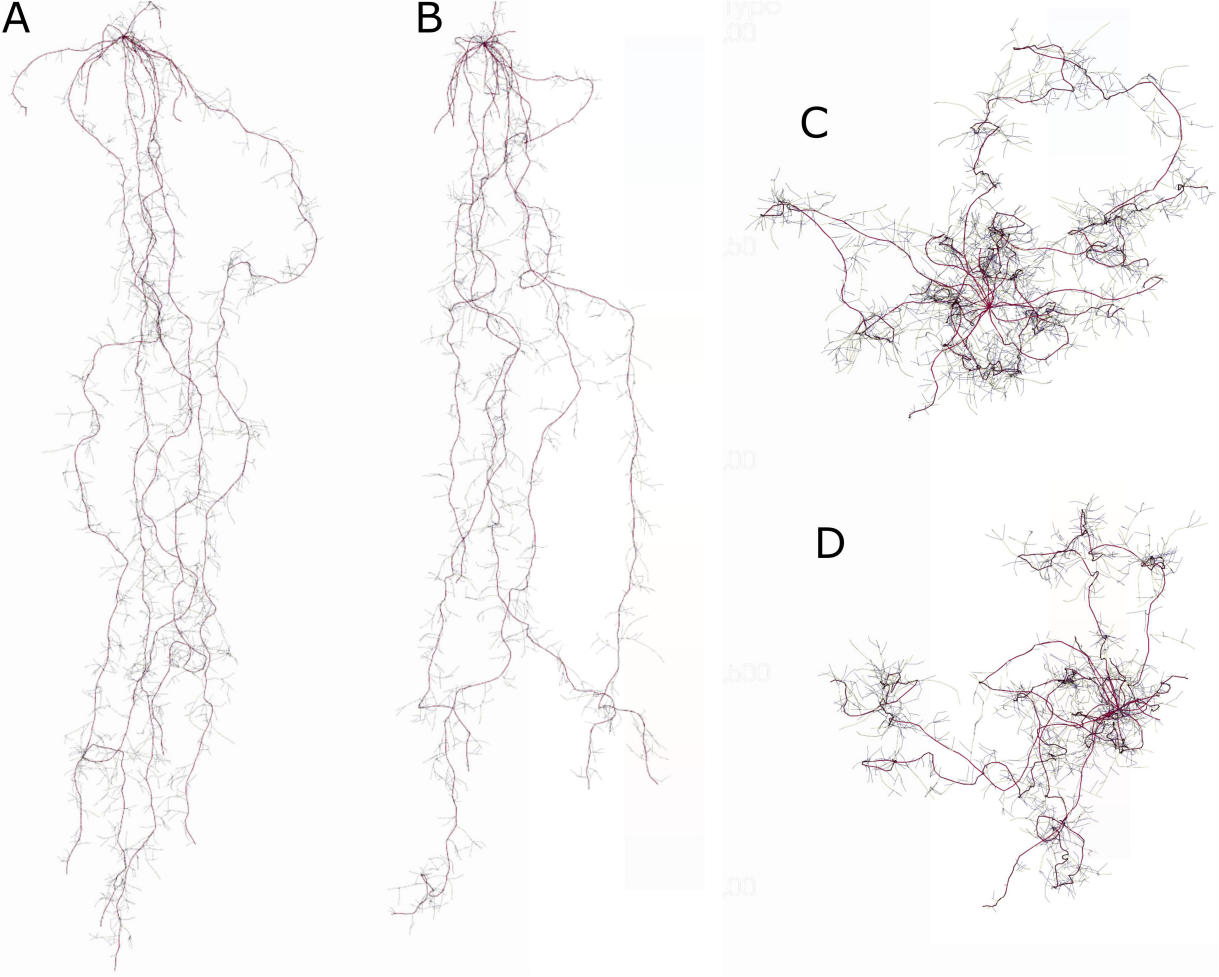
Simulated winter wheat root system (treatment DL) at flowering in 2019/20 for the default ln parameter of 2 [side view **(A)** and top view **(C)**] and for a high ln parameter of 2.5 [side view **(B)** and top view **(D)**] using the coupled SIMPLACE-CRootbox model. Extension of root systems: to 183 from soil depth for ln = 2 and 0–180 cm from soil depth for ln = 2.5 cm. Red color: root of first order, blue color: root of second order.

The simulated grain yield ranged from 6.7 to 6.8 t ha^−1^ for winter wheat and from 5.4 to 6.6 t ha^−1^ for spring barley. The grain yield of virtual phenotypes of both cereals was similar for most situations, although spring barley yield was lower in the control than in the DL treatment. In the case of the control, a high spacing between lateral branches as well as a low initial tip elongation rate decreased winter wheat grain yield by about 0.1 t ha^−1^. The greatest grain yield difference was simulated in spring barley in the control treatment for varying initial tip elongation rate values. Spring barley yield was about 0.2 t ha^−1^lower for the phenotype with a low initial tip elongation rate for the default and enhanced elongation rate phenotypes (control). Also, low number of maximal basal roots reduced spring barley grain yield by about 0.1 t ha^−1^ (control). No yield differences for varying virtual phenotypes were found in the DL treatment. Although the mean RLD was more than 30% higher for the virtual phenotype with the high spacing between lateral branches than for the phenotype with default value (Figure 5), the simulated winter wheat yield difference was only 1%.

The parameter with the greatest influence on yield (Figure 4), initial tip elongation rate r, was further tested under the twelve-year weather data. In the case of winter wheat, a low r value of 5 resulted in a slightly higher effect of DL on mean grain yield (3% of yield gain due to deep loosening as compared to control) compared to the default r value (2 % of yield gain due to DL as compared to control). The high r value resulted in a slightly higher mean winter wheat yield and an effect of DL on mean grain yield of 2 %.The variation of the parameter r showed a higher impact on the simulated yield of spring barley. A low initial tip elongation rate r resulted in lower yields with mean a grain yield of 5 (*r* = 9) and 4.7 t ha^−1^(*r* = 5) in the control. The yield difference due to DL increased from 8% (*r* = 9) to 10% (*r* = 7) to 13% (*r* = 5).

## Discussion

### Assessment of the Observed Impact of Deep Loosening on Root Growth and Yield

Schneider and Don (2019) defined root-restricting layers in German agricultural soils including soil compactness based on a literature review. The authors classified soil bulk densities ranging from 1.75 to 1.82 g cm^−1^ as moderate rooting barriers and soil bulk densities above 1.82 g cm^−1^ as severe barriers for rooting. With values ranging from 1.20 to 1.61 g cm^−3^, the soil bulk densities of our field are even lower than the threshold for moderate rooting barrier. However, roots elongate more slowly in dry soils (with enhanced soil strength) because of a combination of drought stress and mechanical impedance (Bengough et al., 2011), which appeared in the drought spell in 2018.

In 2018, the yield of spring barley was unusually higher than that of winter wheat. An explanation for this is could be sufficient soil water availability in spring, with 100 mm from January to March for winter wheat leading to a rather shallow rooting system but a severe dry spell that occurred afterward and especially during the flowering of winter wheat at the end of May 2018 (rainfall in May: 47 mm). In 2018, the level of winter wheat yield was very low because of the dry spell and heat the cereals faced in that growth period, which caused emergency ripening.

Although the strip-wise DL led to enhanced RLD of both kinds of cereal in most soil depths at the strip in the DL treatment as compared to the control, no significant yield effects were observed. Similarly, such compensatory root growth behavior in the loose soil was observed by Pfeifer et al. (2014). The authors used vertically divided split-root rhizotrons filled either uniformly with loose or compacted peat, or heterogeneously with loose peat in one compartment and compacted peat in the other to assess the impact of soil compaction on root system architecture and root growth dynamics of barley under sufficient water supply in a controlled-environment chamber. They observed greater root length, earlier occurrence of laterals, and larger root area of roots under loose soil than under uniformly loose conditions or in all compacted compartments (Pfeifer et al., 2014). The RLD was highest for the treatment filled uniformly with loose peat (Pfeifer et al., 2014).

In a meta-analysis, Schneider et al. (2017) evaluated the effect of deep tillage on crop yield and reported that on average, deep tillage slightly increased yield (+6%). This is in line with our weather scenario simulation runs where a mean grain yield increase of 10% for spring barley and 2% for winter wheat for treatment DL as compared to the control was simulated. The individual deep tillage effects were however highly site-specific, and about 40% of the considered studies reported yield depression after deep tillage (Schneider et al., 2017).

### What Do We Gain From the Modeling Study?

The effect of a given level of compaction is related to both weather and crop species, since soil strength is a function of soil compaction and soil moisture (Batey, 2009). The effects of soil compaction are, thus, greater under warmer and dryer climates, especially when dense layers impede access to water in deeper soil layers. We used site-specific values of soil texture, organic carbon content, and soil strength (as a function of soil matrix potential and bulk density) along with crop management information (sowing date, crop development, with/without deep loosening) to simulate shoot and root growth. The increased absolute RLD in DL treatment compared to control was predicted by the SIMPLACE-CRootbox model in most cases but to a lesser extent than observed. Since estimation of the absolute RLD *via* the profile wall method and the monolith factor is also not exact and RLD depends not only on penetration resistance but also on other small-scale factors (soil water heterogeneity, nutrient hot spots, and barriers for roots such as stones) that were not simulated, these deviations are acceptable. Thus, model simulations can be conducted to improve our understanding of how often and under which conditions subsoil loosening leads to positive yield effects at that site. The simulations from 2017 to 2019 at the experimental site indicate that the pronounced early root development of spring barley due to reduced soil strength in the DL treatment was favorable for crop growth at least in years with drought stress. This was also observed in the first sampling date in 2017 from 0 to 60 cm soil depth. These results highlight the importance of early root development of spring cereals.

Similar simulated root biomass but higher cumulative RLD in the DL compared than in the control treatment points to thinner roots with smaller root diameters in treatment DL especially in 2018 (Supplementary Figure 7). In 2018, the observed average root diameter of spring barley was 0.29 mm (control) vs. 0.25 mm (DL), and for winter wheat it was 0.31 mm (control) vs. 0.3 mm (DL) from 40 to 60 cm soil depth. These observations support the simulation results of thinner roots in the DL treatment (with higher soil water holding capacity) compared to the control. This finding is in line with Correa et al. (2019), Popova et al. (2016), and Pfeifer et al. (2014) who have shown that root diameter was increased in compacted soil, since thicker roots have a greater ability to explore hard soil, as well as with Ahmadi et al. (2018) who reported that roots become thicker as soil water deficit increases. Goss (1977) demonstrated that when only the apical parts of main root axes of barley plants are subjected to soil compaction, the lateral roots that penetrate freely into loose soil have a much greater length than the lateral roots of plants whose root systems grow completely unimpeded. This increased lateral root growth could mask the effects of compacted soil on the main root axis if the total dry root mass is similar between the unaffected and the impeded root main axes (Goss, 1977). This could be similar in our case, since the simulated total root dry mass was similar in both treatments in most growth periods (except for spring barley 2018). Thus, enhanced RLD in the DL treatment may be caused by thinner roots rather than by more roots leading to similar shoot growth.

### Is the Model Able to Represent the Behavior of Different Virtual Root Phenotypes?

The root phenotype is very plastic and influenced by numerous interactions. Since metabolic costs of enhanced root growth are relatively high, breeding for genotypes having an increased allocation of resources to roots may carry negative consequences for yield, especially in resource-poor environments (Lynch, 2007; Mi et al., 2010; Correa et al., 2019). Thus, it is important to quantify G × E effects for the selection of appropriate cultivars for a specific site as well as for breeding. In general, shallow rooting genotypes are vulnerable to soil compaction, especially under rain-fed conditions when a plant relies on water from deeper soil layers. In our field trial CF1-3 in 2019/20, we did not observe significant grain yield differences among three cultivars of spring barley and winter wheat with different root growth patterns.

We varied three CRootbox parameters for testing the effect of different virtual but plausible phenotypes close to our field observations on simulated root and shoot growth. By simulation-based testing of different root phenotypes with various RLD distributions over soil depth, we were able to quantify the effects of different virtual root phenotypes as well as of subsoil loosening. The most sensitive parameter was initial tip elongation rate r and, again, highlights the importance of early root development of spring cereals.

CRootbox is capable of representing the behavior of different root phenotypes, which is a clear improvement compared to SIMPLACE-SlimRoots. Such models may support future root phenotyping by indicating the importance of specific root phenes. They will also help to better understand the benefit of root phenotypic plasticity as a breeding target for crops in variable environments (Schneider and Lynch, 2020).

### Model Comparison and Improvement

The two applied root models, SlimRoots and CRootbox, are comparable to some extent only. The two approaches follow different concepts: while SlimRoots is a simple rule-based model, CRootbox is a flexible functional-structural root model that is based on state-of-the-art computational science methods and is driven by carbon availability, local root age, and local soil conditions. However, prioritization of seminal roots was not (yet) implemented for CRootbox. With the SIMPLACE-CRootbox model, the best of both worlds was combined: the more detailed 3D root model provides information on root traits while it benefits from the consideration of the soil-plant-atmosphere system (e.g., feedback to shoot and soil water dynamics) and the computational efficiency of the 1D soil-crop model.

On the one hand, the coupled SIMPLACE-CRootbox model can be interpreted as an extension of CRootbox by adding the whole crop modeling part. This allows for the consideration of the feedback to the shoot *via* carbon allocation to the roots as well as simulation of yield. This hybrid 1D/3D model also overcomes the drawback of the extensive computational effort of fully 3D functional-structural plant (FSP) models (Evers et al., 2018) and allows for, e.g., scenario analysis.

On the other hand, the coupled model can be interpreted as an improvement of the 1D field-scale modeling platform SIMPLACE by enabling 3D root architecture development with effects of local soil conditions experienced by each root tip on the resulting structure without greatly increasing the computational effort. The study showed improved simulation of root growth considering soil strength with SIMPLACE-cRootbox as compared to SIMPLACE-SlimRoots. SIMPLACE-SlimRoots with the simple root model was not able to predict the different rooting pattern and resulting yields caused by subsoil loosening. For studies focusing on root growth under varying soil conditions and on testing different root growth patterns, 1D field-scale models coupled with 3D root architecture models such as the presented SIMPLACE-cRootbox model are recommended.

In the future, more extended coupling could make use of CRootbox' ability to compute not only the RLD in each soil layer but also root water uptake sink terms for each soil layer based on root hydraulic architecture.The common variable of both models would be root water uptake per layer, and CRootbox would not only pass the RLD but also the root water uptake to SIMPLACE and get the soil matrix potential in each layer from the SIMPLACE soil component beforehand. This will enable to include further processes not accounted for in the current simulations, i.e., root water uptake compensation and recognition of local root hydraulic properties (Meunier et al., 2019). Further field data (more seasons per cultivar, more sites) would allow for more robust model calibration. For that study, usage of default parameter values from widely used models (such as Lintul-2) with few parameter adaptations to account for cultivar specific traits was feasible. The calibration aimed to simulate i) anthesis and maturity dates that affect daily root growth and biomass increment, ii) the main pattern of RLD over time and depth, and iii) the shoot biomass that drives water and nutrient uptake. Usage of an algorithm for calibration seemed unnecessary for that. Furthermore, correct simulation of soil water dynamics is important, and measurements of soil water content in several soil depths over time would be favorable. The simulation of soil settlement after deep loosening over time should be considered in further (long-term) simulation studies.

## Conclusions

An enhanced RLD due to deep loosening may lead to a significant effect on spring barley yield at the presented experimental site under certain weather conditions. Further field data with and without dry spells and soil layers with and without compaction as well as different species and cultivars would provide more data to enhance the accuracy and robustness of model predictions.

We conclude that such 1D/3D models are feasible tools to assess the value of physiological traits in targeted environments and for specific management (genetics × environment × management, G × E × M) for informed choice of cultivars or to focus research on promising root traits (*in silico* breeding). In agreement with Postma et al. (2013), we assume that the cycles of model-based hypothesis generation and derivation of rules by evaluation of simulations and their experimental verification must be prioritized in the field of root research. An *in silico* exploration of GxExM interactions where crop models can be used to simulate the impact of plant traits on yield has the advantage that sample sizes, numbers of replications, numbers of factors, and modifications can be very large, and that the time required to conduct an *in silico* experiment is very small. This allows for evaluation of a large number of possible G × E × M combinations (scenarios). However, such an approach has to be combined with real experiments for model calibration and validation.

## Data Availability Statement

The original contributions presented in the study are included in the article/Supplementary Material, further inquiries can be directed to the corresponding author/s.

## Author Contributions

SS, AS, TG, and FE contributed to conception and design of the study. MA, TK, JG, and OS designed the field trial and collected the data. SS wrote the first draft of the manuscript. AS and DL helped with the writing and coding/model coupling. All authors contributed to manuscript revision, read, and approved the submitted version.

## Funding

This study was funded by the BonaRes project Soil3 (BOMA 03037514, 031B0515C) of the Federal Ministry of Education and Research (BMBF), Germany, and by Deutsche Forschungsgemeinschaft (DFG, German Research Foundation) under Germany's Excellence Strategy-EXC 2070-390732324.

## Conflict of Interest

DL was employed by Simulationswerkstatt. The remaining authors declare that the research was conducted in the absence of any commercial or financial relationships that could be construed as a potential conflict of interest.

## Publisher's Note

All claims expressed in this article are solely those of the authors and do not necessarily represent those of their affiliated organizations, or those of the publisher, the editors and the reviewers. Any product that may be evaluated in this article, or claim that may be made by its manufacturer, is not guaranteed or endorsed by the publisher.
