# Supplementary material for: Simulating Root Growth as a Function of Soil Strength and Yield With a Field-Scale Crop Model Coupled With a 3D Architectural Root Model
Source: Front Plant Sci. 2022 May 20;13:865188. doi: 10.3389/fpls.2022.865188 (PMC9164166; doi:10.3389/fpls.2022.865188)
Supplement: Supplementary file 1 [file Data_Sheet_1.PDF]

Supplementary Material

Article title: Modeling root elongation as a function of soil strength and biomass growth with a field-scale crop model coupled with a 3D architectural root model

Supplementary Table 2. Applied LintulPhenology and LintulBiomass parameter values (van Oijen and Leffelaar, 2008; www.simplace.net/doc) and CRootBox parameter values per root type and plant parameters needed for the root architecture development (Schnepf et al., 2018b; Morandage et al., 2019; Morandage et al., 2021). In case the parameters deviate from the default values, the default values are given in brackets (if available). T sum stands for temperature sum. For root nomenclature see Zobel and Waisel (2010).

**parameter**	**unit**	**explanation**	**spring barley**	**winter wheat**
AirTemperatureSumAnthesis	°C day	T sum from emergence to anthesis	680 (675)	400
AirTemperatureSumMaturity	°C day	T sum after anthesis to physiological maturity	1160	1200 (1350)
LUE	g MJ^{-1}	light use efficiency	2.5	2.4 (3.0)
specific root weight	g cm^{-1}	[required to couple both models]	0.00002	0.00002
		**CRootBox taproot parameters**		
a	cm	root radius	0.0325	0.055
r	cm d^{-1}	Initial tip elongation rate	7 (1.68)	7 (1.2)
la	cm	Length of apical zone	3.2	4.2
lb	cm	Length of basal zone	2.8	0.8
ln	cm	Length between lateral branches	0.85 (1.099)	2 (1.2)
lmax	cm	Maximal root length	160 (150)	210 (130)
firstB	day	First emergence of basal roots	1	60
delayB	day	Time period between basal roots	14	9
maxB	1	Maximal number of basal roots	5	20
firstS	day	First occurrence of shoot-borne roots	140	140
delayS	day	Time period between shoot-borne roots	7	20
nC	cm	number of shoot-borne roots per root crown	0	0
		**CRootBox first order lateral root parameters**		
a	cm	root radius	0.0115	0.03
r	cm d^{-1}	Initial tip elongation rate	0.3	0.4
la	cm	Length of apical zone	0.713	1.8
lb	cm	Length of basal zone	0.452	0.8
ln	cm	Length between lateral branches	2.0	1.1
lmax	cm	Maximal root length	4.8	5.0
		**CRootBox second order lateral root parameters**		
a	cm	root radius	0.025	0.02
r	cm d^{-1}	Initial tip elongation rate	0.1	1
la	cm	Length of apical zone	1.5	2.2
lb	cm	Length of basal zone	0	0
ln	cm	Length between lateral branches	0	0
lmax	cm	Maximal root length	1	0.56

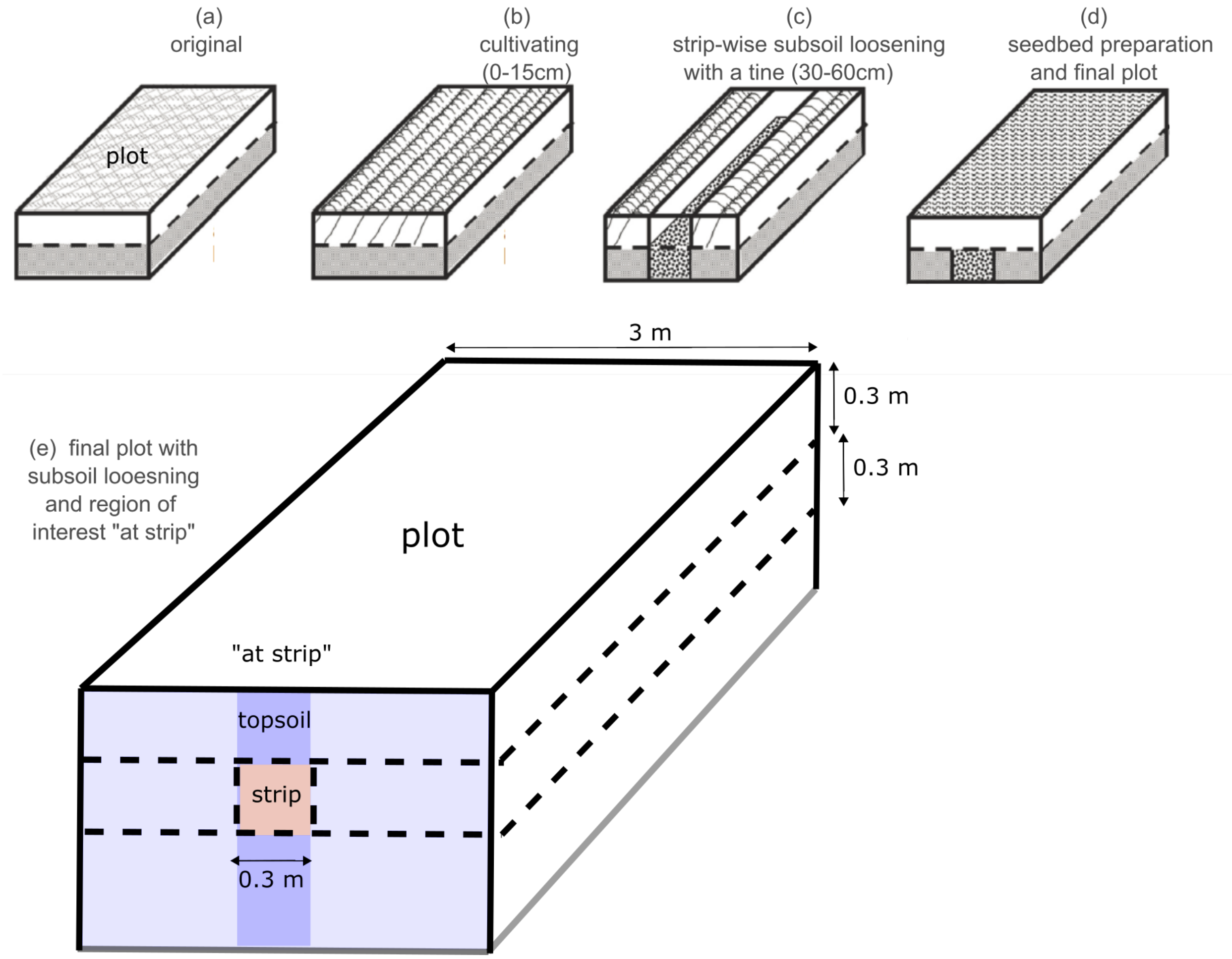

Supplementary Figure 1. Scheme of the strip-wise subsoil loosening at CF1 CKA. Preparation included the removal of uncontrolled growth (a), followed by ploughing up to 0.3 m depth (b). The strips at 30 to 60 cm soil depth were prepared using a tine (c). The topsoil was laid back and reconsolidated. Disc harrowing was applied for seedbed preparation (d). (e) shows the region of interest "at strip" of a plot (treatment with deep looseing, DL). The scheme was taken from Jakobs et al. (2017) and modified.

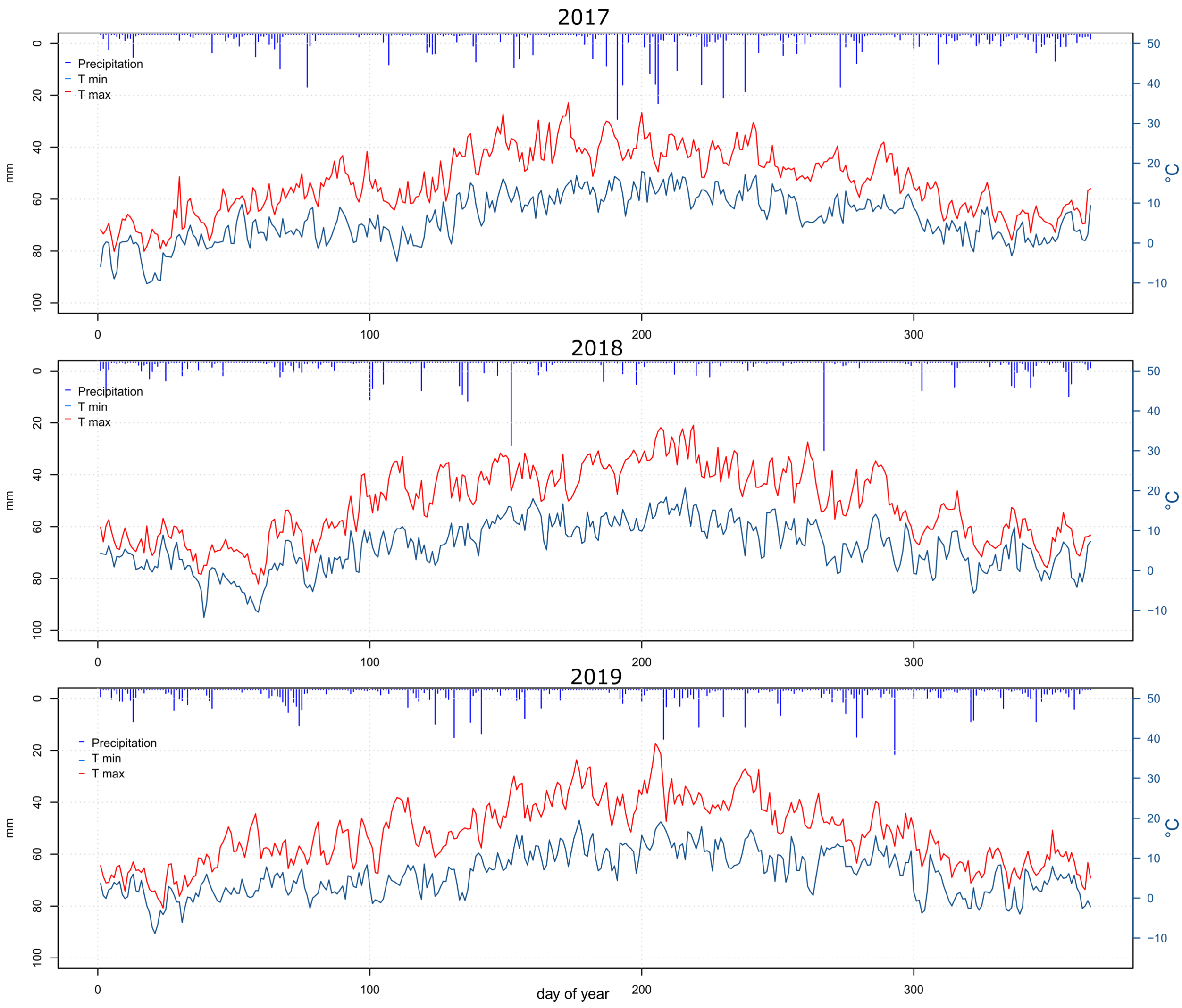

Supplementary Figure 2. Daily precipitation (mm) and daily minimum and maximum air temperature (T min and T max, both in ° C) in 2017, 2018 and 2019 at the experimental station Campus Klein-Altendorf (University of Bonn), Germany.

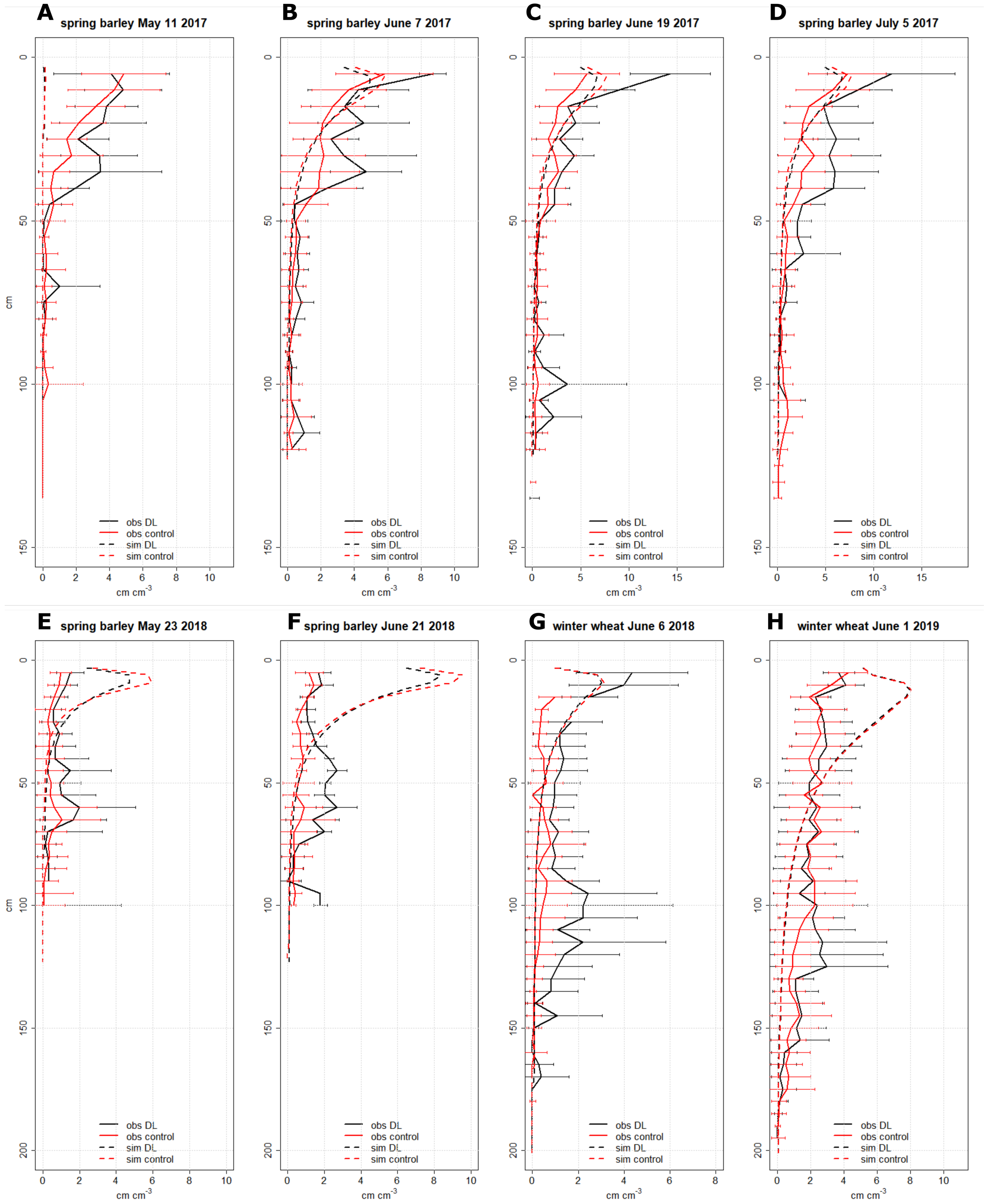

Supplementary Figure 3. Observed (mean and standard deviation) and simulated (SIMPLACE-SlimRoots) absolute root length densities over soil depth in cm cm^{-3} over soil depth in cm of spring barley (2017 and 2018) and winter wheat (2018 and 2019) for the control and the DL (at strip) treatment. The RLD data was observed in profile walls and converted to absolute values based on profile wall and monolith data. The RLD data observed with the profile wall method was partly published in Jakobs et al. (2019).

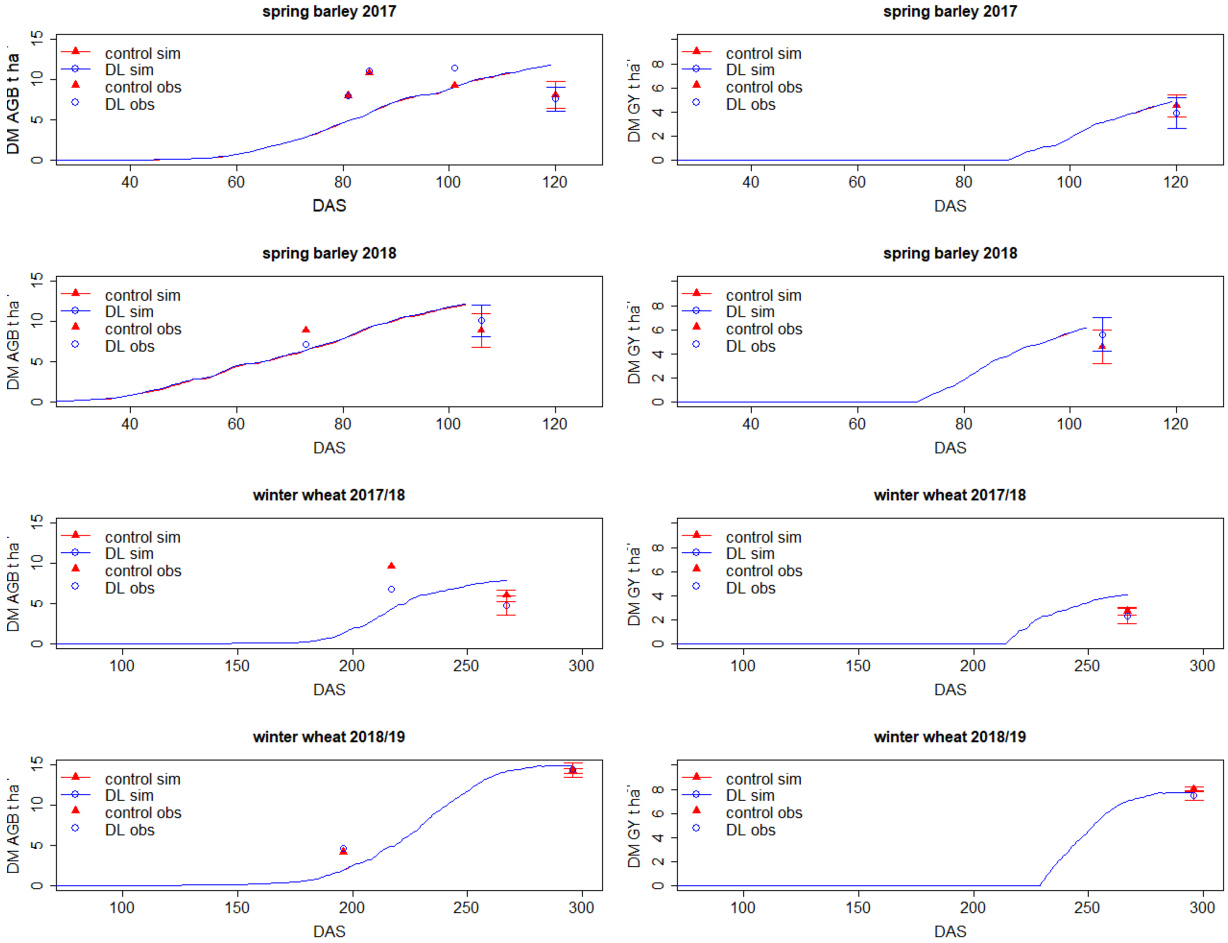

Supplementary Figure 4. Observed and simulated (SIMPLACE-SlimRoots) dry matter above-ground biomass during the season and at harvest (DM AGB) and grain yield (DM GY) in t ha^{-1} of spring barley (2017, 2018) and winter wheat (2017/18 and 2018/19) for the control and the DL (at strip) treatment. The yield data at harvest is given as mean values with standard deviation.

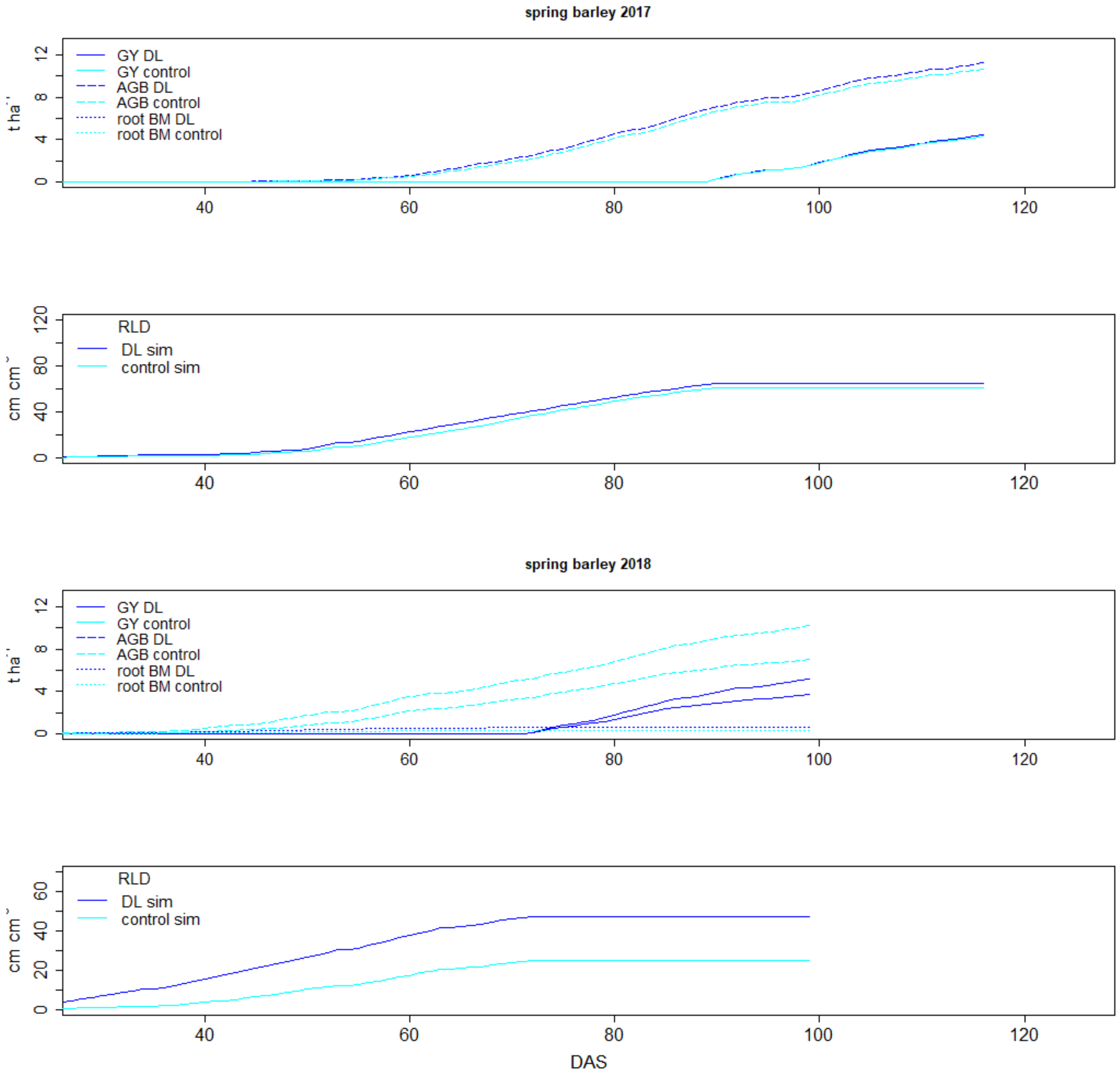

Supplementary Figure 5. Simulated daily grain yield (GY), above-ground biomass (AGB) and root biomass (root BM) in t ha^{-1} and cumulative root length density (RLD) in cm cm^{-3} over soil depth of spring barley (2017, 2018) for the control and the DL (at strip) treatment using the coupled model SIMPLACE-CRootBox.

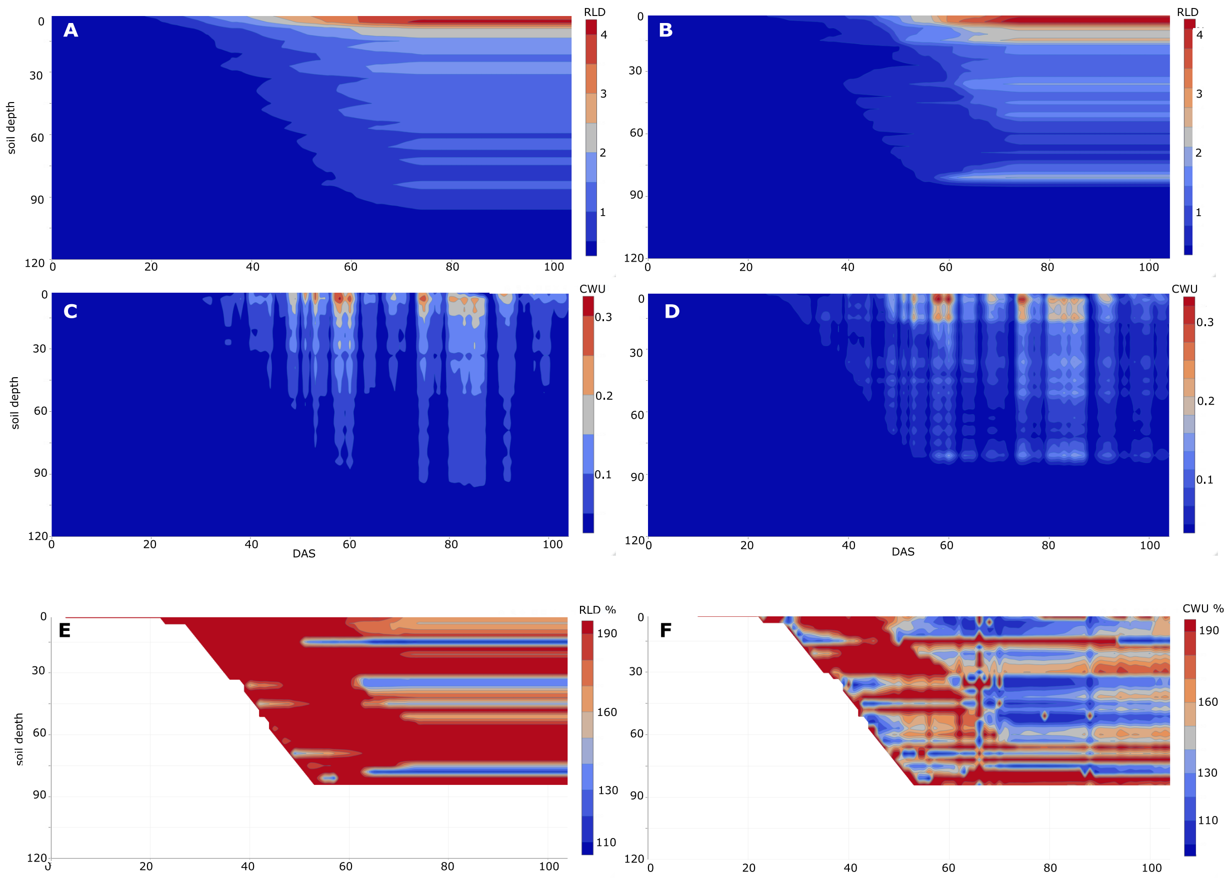

Supplementary Figure 6. Simulated daily root length density (RLD, panels A and B) in cm cm^{-3} and crop water uptake (CWU, panels C and D) in mm over soil depth of spring barley (2018) for the DL (at strip, left panels A and C) and the control (right panels B and D) treatment using the coupled model SIMPLACE-cRootBox. The cumulative CWU was 124 mm (DL) and 81 mm (control). Panels E and F show the differences in RLD and CWU between both treatments in % (control=100%).

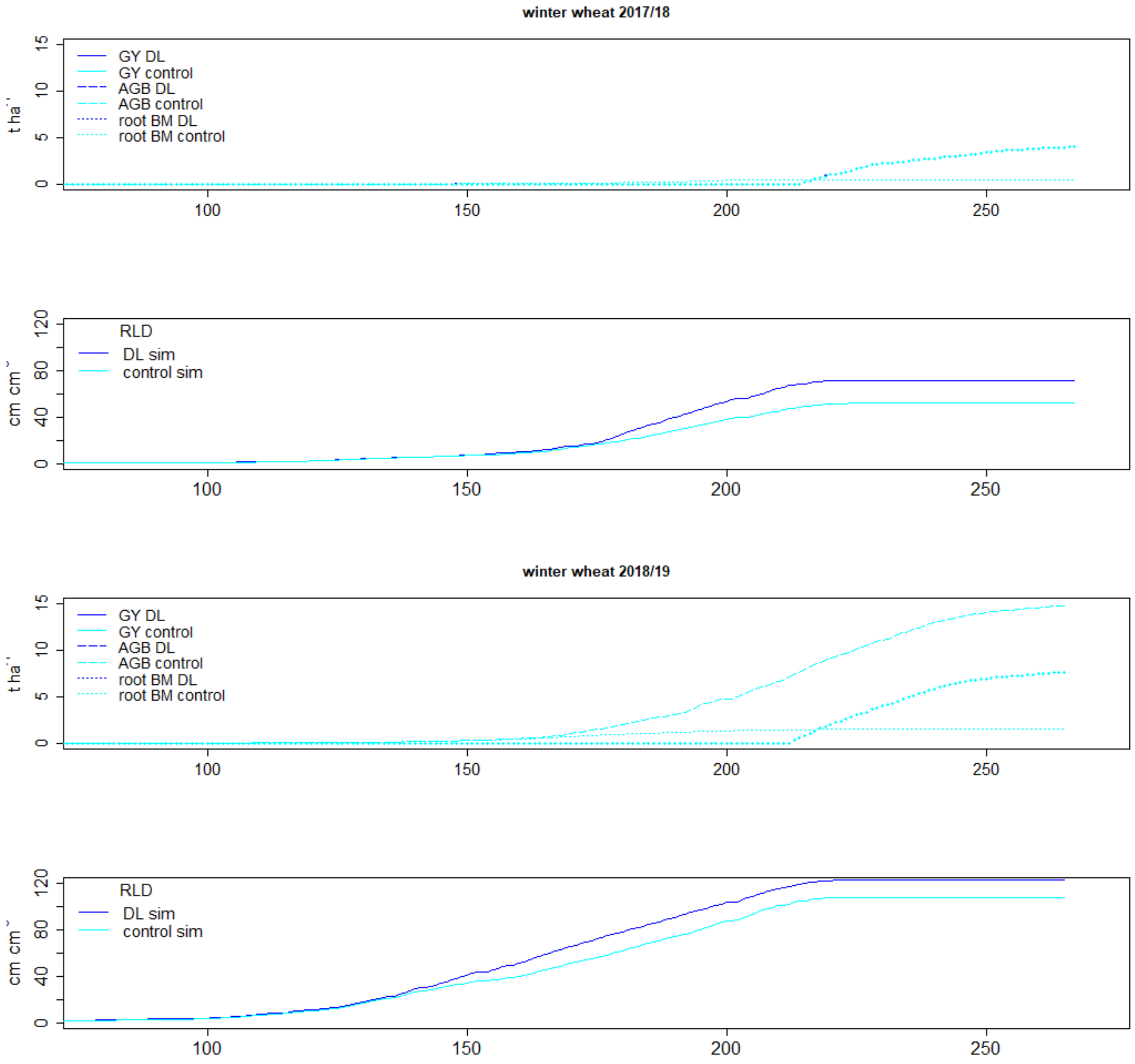

Supplementary Figure 7. Simulated daily grain yield (GY), above-ground biomass (AGB) and root biomass (root BM) in t ha^{-1} and cumulative root length density in cm cm^{-3} over soil depth of winter wheat (2017/18 and 2018/19) for the control and the DL (at strip) treatment using the coupled model SIMPLACE-cRootBox.

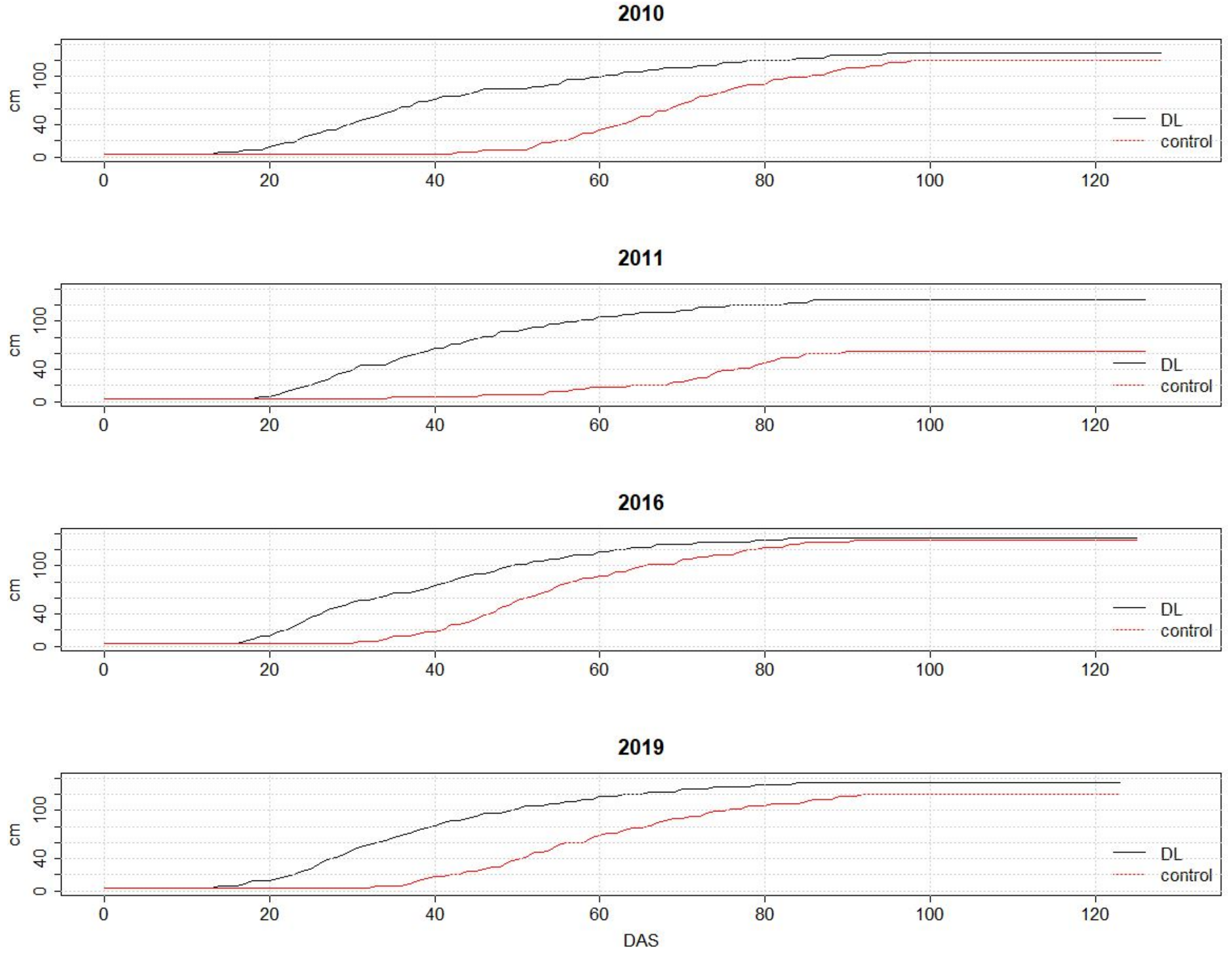

Supplementary Figure 8. Simulated daily maximal spring barely rooting depth (cm) of the control and of the DL (at strip) treatment for four selected seasons (2010, 2011, 2016, 2016) of the weather scenario simulation runs using the coupled model SIMPLACE-cRootBox. DAS stands for days after sowing.
